# Adsorptive removal of Cr(VI) ions using nitrogen-doping activated carbon: influence of pH, kinetics, isotherm models, optimization, and efficiency evaluation

**DOI:** 10.1038/s41598-026-53699-7

**Published:** 2026-06-11

**Authors:** Mohamed A. El Nemr, Hossam S. Jahin, Mohamed H. H. Abbas, Mohamed A. Hassaan, Mohamed N. M. Ismail, Ahmed El Nemr

**Affiliations:** 1 Department of Chemical Engineering, Faculty of Engineering, Minia University, Minia, 61519 Egypt; 2 The Higher Canal Institute of Engineering and Technology, Al Salam 1-Abu Bakr Al Siddiq Street, Suez, Egypt; 3 Central Laboratory for Environmental Quality Monitoring, National Water Research Center, El-kantir, 13621 Egypt; 4 Department of Biotechnology, Faculty of Applied Health Science, October 6th University, October 6th City, 12585 Egypt; 5 Soils and Water Department, Faculty of Agriculture, Benha University, Benha, Egypt; 6 National Institute of Oceanography and Fisheries (NIOF), Kayet Bey, Elanfoushy Alexandria, Egypt

**Keywords:** Activated carbon, Hexavalent chromium, Removal efficiency, RSM model, Sorption isotherms, Sorption kinetics, Chemistry, Environmental sciences, Materials science

## Abstract

Hexavalent chromium ion [Cr(VI)] poses serious environmental risks due to its high toxicity and ability to cross biological membranes, leading to organ damage and genetic mutations. This study presents a new practical method for removing Cr(VI) ions from wastewater using nitrogen-doped activated carbon (AC600) prepared by pyrolyzing sawdust with ZnCl_2_ at 600 °C. Thereafter, batch experiments were conducted using different adsorbent doses (0.5–2.5 g L^–1^) and Cr(VI) ion concentrations (100–400 mg/L) at pH ranging from 1 to 12. Results revealed that Cr(VI) ions sorption on AC600 followed both linear and adsorption isotherm models at low sorbent doses (< 1.5 g/L). At higher doses, sorption shifted toward a hyperbolic adsorption pattern. Overall sorption data fitted well with both the Freundlich and Temkin isotherm models. Cr(VI) ions removal efficiency increased with contact time, following a sharp rise within the first 20–40 min of contact, while equilibrating within approximately 60–80 min. However, this efficiency decreased as pH increased. The highest efficiency (95–99%) was achieved using 2.5 g of AC600 to treat 1 L of water containing 100–150 mg/L of Cr(VI) ions within 2 h. For higher Cr(VI) ion concentration (200–250 mg/L), the efficiency decreased to 88–90%. The kinetics of Cr(VI) ion sorption on activated carbon followed the Pseudo-Second-Order model at low to moderate AC600 doses and low Cr(VI) ion concentrations. At higher doses and Cr(VI) levels, the power function and intraparticle diffusion models provided better fits. A maximum adsorption % could be achieved by using 2.5 g of AC600 and a Cr(VI) ion solution at 100 mg/L, according to results from an optimization of adsorption parameters using response surface methodology (RSM).

**Supplementary Information:**

The online version contains supplementary material available at 10.1038/s41598-026-53699-7.

## Introduction

Chromium exists in various oxidation states (from 2− to 6+), but only two forms are present in nature: Cr(III) and Cr(IV) ions^1^. Although, Cr(III) is an essential micronutrient for plants, hence Cr(VI) ions exhibit high toxicity^2,3^ due to their high capability to penetrate biological membranes, causing serious health issues such as organ damage, oxidative stress, and genetic alterations in plants and animals^4^. The toxicity of Cr(VI) is about 300 times higher than that of Cr(III)^1^. This contaminant persists in water, making its cleanup difficult and thus impacting ecosystems and aquatic life (Tasnim et al., 2024).

Growing environmental and health concerns regarding Cr(VI) ion contamination^5^ have prompted a search for sustainable, cost-effective treatment methods^6^. One promising technique is the use of carbon-based adsorbents, made from natural or waste materials^7^, for water decontamination. These materials are produced from low-cost materials via pyrolysis of organic carbon-rich biomass^8^ and have several advantages, such as low production cost, renewable feedstocks, and a highly porous structure that exhibits its high sorption capacity^9^ for contaminants in soil and water^8^. For example, activated carbon-based systems demonstrated high removal efficiencies of Cr(VI) ions as high as 93–95.97%^9,10^.

In this study, nitrogen-doped activated carbon was produced from sawdust (an abundant precursor) using a chemical activation and pyrolysis process, which will be evaluated to observe its possible effect on the removal efficiency of Cr(VI) ions from wastewater and speed up this process. This eco-friendly and cost-effective procedure increases the total functional groups, particularly –NH_2_ ones. In this regard, amino groups which are considered hard base synergistically chelate Cr(VI) ions (hard acid)^11^ according to the concepts of the HSAB (Hard and Soft Acids and Bases) theory, stating that hard acids prefer hard bases, while soft acids prefer soft bases. This modification may effectively remove more Cr(VI) ions from contaminated waters. Therefore, N-doped activated carbons was synthesized from sawdust using ZnCl_2_ as an activating material through pyrolysis at 600 ºC (AC600). The resulting product was used, at different doses, to remove various concentrations of Cr(VI) ions, while assessing Cr(VI) removal efficiency, adsorption kinetics, and overall adsorption behavior on the activated carbon^12,13,14^. In addition, response surface methodology (RSM) and ANN optimization of Cr(VI) ions removal from water were conducted to provide a novel approach that combines material development with enhanced Cr(IV) removal efficiency. This work supports several Goals: (1) removal of Cr via recycling wastes, produced from low-cost, eco-friendly adsorbent; hence protect the environment from waste disposal.

## Materials and methods

### Materials of study

Sawdust was collected from a local carpenter’s workshop in Alexandria, Egypt. The sawdust was dried in an oven at 105 °C for 24 h to a constant weight, and the particle size was standardized using sieving 1–2 mm to ensure uniform processing. Hydrochloric acid (HCl, MW 36.46 g/mol and an assay of 30–34%) was purchased from SD Fine-Chem Ltd (SD FCL), Mumbai, India. Zinc chloride (ZnCl_2_, reagent grade, ≥ 98%) and potassium dichromate (K_2_Cr_2_O_7_, M.W. 294.19 g/mol, assay 99%) were obtained from Sigma-Aldrich. For spectrophotometric detection of hexavalent chromium, 1,5-diphenylcarbazide was purchased from BDHZ Chemicals Ltd., Poole, England.

### Preparation of nitrogen-doping activated carbon

100 g of sawdust was mixed with 50 g ZnCl_2_ in 300 mL of distilled H_2_O. The mixture was placed in a 500 mL handmade Teflon cup that was placed into a stainless-steel autoclave and hydrothermally treated for five hours at 180 °C. The product was transferred to a mortar, dried at 125 °C overnight, and then carbonized at 600 °C under flow of NH_3_ gas (100 mL/min) for 1 h (The experimental setup used for NH_3_ activation, specifying the use of a tube furnace, quartz boat, and gas flow meter to control the reaction environment, enhancing understanding of the process). The carbonized material was refluxed for 2 h in 2 N HCl (to remove ash) to remove residual contents, filtered, and dried overnight at 125 °C. This was followed by sonication (activation and pores purification) in H_2_O for 0.5 h, decantation, then washing with ethanol (Cleaning and drying the AC600) and oven drying at 125 °C overnight. The nitrogen-doping activated carbon product was designated as AC600.

### Characterization

For Cr(VI) ion concentration measurement, an Analytic Jena (SPEKOL1300 UV/Visible spectrophotometer) with a 1 cm optical glass cell path was employed. Thermo shaker incubator (GSSI-100T sh), tubular furnace Nabertherm B180 (RT 50/250/13), JENCO (6173) pH meter, and shaker [A JS shaker (JSOS-500)] were utilized for the experiment. The functional groups and surface chemical state of the Nitrogen-Doped Activated Carbon (NDACs) were ascertained using a Fourier transform infrared spectrometer (FT-IR: Bruker Vertex 70 connected to Platinum ATR model V-100). The morphology of the NDACs was examined using an EDX device and a scanning electron microscope (SEM: LEO, 1450VP). The volume of monolayer (*V*_m_) (cm^3^ (STP) g^–1^), the surface area (*S*_BET_) (m^2^/g), volume of total pores (*V*_T_) (*p*/*p*_0_) (cm^3^/g), energy constant (*C*), mean diameter of pores (nm) and the average pore radius were calculated according to BET^15^ analysis of the isotherm. N_2_ adsorption at 77 K was used to get the BET surface area (*S*_BET_) study of the NDACs using an analyzer device (BELSORP – Mini II, BEL Japan, Inc.)^16,17^. Additionally, using the BELSORP analysis program software, the Barrett-Joyner–Halenda (BJH) techniques were used to calculate the surface area of the micropore (*S*_mi_) and volume of the micropore (*V*_mi_), as well as the surface area of the mesopore (*S*_mes_) and volume of the mesopore (*V*_mes_) of NDACs. Using the BJH approach, the distribution of pore size was determined from the desorption isotherm^15^. Using the SDT650-Simultaneous Thermal Analyzer device, thermal analyses were performed to determine the thermal stability at temperatures ranging from 25 to 1000 °C with a temperature ramp rate of 10 °C/min under a nitrogen gas flow rate of 100 mL/min^2^. The XRD analysis was performed using a D2 PHASER Instrument produced by Bruker in Germany^18^. A Thermo Fisher Scientific K-Alpha XPS with a pass energy of 50 eV and a base pressure of around 10^–9^ mbar was used to perform elemental analysis.

### Preparation of Cr(VI) stock and working solutions

A stock solution of 1000 mg L^–1^ of Cr(VI) ion was prepared by dissolving 2.8289 g of K_2_Cr_2_O_7_ (analytical grade) in a liter of double-distilled water, then this stock was diluted to prepare the different concentrations of Cr(VI) ions, ranging from 100 to 400 mg L^–1^.

### Experimental procedure

Batch experiments were conducted under lab conditions using 100 mL of Cr(VI) ion solution at room temperature (25 ± 2 °C) in 300 mL Erlenmeyer flasks to evaluate the adsorption capability of AC600 for removing Cr(VI) ion from artificially contaminated waters. Synthetic Cr(VI) solutions were prepared in deionized water, which is standard for initial performance evaluation. To attain this aim, batch experiments were followed by mixing various adsorbent doses (0.5–2.5 g L^–1^) with different initial Cr(VI) ions concentration (100–400 mg L^–1^); then water samples (0.5 mL) were collected at the following periods: 10, 15, 30, 45, 60, 90, and 120 min. Samples were shaken at 200 rpm using an orbital shaker, filtered through 0.45 μm membrane filters, and then the Cr(VI) ion concentration was analyzed in the filtrate by UV–Vis-spectrophotometer at *λ*_max_ = 545 nm using 1,5-diphenylcarbazide as a reagent^19,18^. Finally, the pH effect was evaluated using a 100 mg L^–1^ Cr(VI) ions solution with 100 mg of adsorbent adjusted at pH 1.5, 3.1, 5.2, 7.1, 9.2 and 11.3 using either 0.1 M HCl or 0.1 M NaOH. All experiments were performed in triplicate at ambient temperature (25 ± 2 °C). The standard deviation of the measured values did not exceed 2.2 in any case, and the mean values were used for all subsequent calculations^20,21,13,22^.

### Analytical procedure

The control and three spikes were assessed to ensure the measurement accuracy of Cr(VI) ions (The Control is defined as a flask containing the Cr(VI) solution without any AC600 adsorbent to check for potential losses due to volatilization or wall effects, confirming its stability. This was a synthetic Cr(VI) solution in deionized water). The amount of Cr(VI) ions adsorbed per unit mass of activated carbon (*q*_e_, mg g^–1^) was calculated using the following Eq. (1).

1 $$\:{q}_{e}=\frac{{C}_{0}-{C}_{e}}{m}\times\:V$$

*C*_0_ and *C*_e_ are the initial and equilibrium concentrations of Cr(VI) ions (mg L^–1^) in suspensions, *V* is the solution volume (*L*), and *m* is the adsorbent mass (g).

Concentrations of Cr(VI) ions at equilibrium (120 min of contact) were also presented graphically versus the corresponding Cr(VI) ions sorbed amount to investigate the mechanisms beyond this process using four adsorption isotherm models, i.e. Langmuir, Freundlich, Temkin, and Halsey isotherm models (HIM) Eq. (2) as outlined by El Nemr et al.^23^, Mishra et al.^24^ and Al-Ghouti and Da’ana^25^.

2 $$\:{q}_{e}=\frac{{q}_{max}{K}_{l}{C}_{e}}{1+{K}_{L}{C}_{e}}$$

*q*_e_ is the sorbed amount per unit mass at equilibrium, *C*_e_ is the equilibrium concentration, *q*_max_ is the maximum adsorption capacity of activated carbon and *K*_L_ is the Langmuir constant.

Freundlich isotherm model (FIM) is represented by Eq. (3):

3 $$\:{q}_{e}={K}_{F}{{C}_{e}}^{\raisebox{1ex}{$1$}\!\left/\:\!\raisebox{-1ex}{$n$}\right.}$$

*K*_F_ and *n* are Freundlich constants related to adsorption capacity and heterogeneity factor.

The Temkin Isotherm Model is represented by Eq. (4)

4 $$\:{q}_{e}=BlnA+Bln{C}_{e}$$

A and B are the Temkin isotherm constants.

Halsey Isotherm equation (HIE) is represented by Eq. (5)

5 $$\:{q}_{e}={K}_{F}{{C}_{e}}^{\raisebox{1ex}{$-1$}\!\left/\:\!\raisebox{-1ex}{$n$}\right.}$$

*K*_F_ = Halsey constant related to adsorption capacity, and “*n*” is another constant related to adsorption intensity or heterogeneity.

Kinetic data were fitted to 4 kinetic models according to El Nemr et al.^23^, Filipović et al.^26^ and Jahin et al.^27^.

Pseudo-first-order is represented by Eq. (6)

6 $$\:{q}_{t}={q}_{e}(1-{e}^{-k1t})$$

*k*_1_ is the pseudo-first-order constant.

Pseudo-second-order models is represented by Eq. (7)

7 $$\:{q}_{t}=\frac{{k}_{2}{q}_{e}^{2}t}{1+{k}_{2}{q}_{2}t}$$

*k*_2_ is the pseudo-second order constant.

Elovich is represented by Eq. (8)

8 $$\:q=\frac{1}{\beta\:}\mathrm{ln}\left(\alpha\:\beta\:\right)+\left(\frac{1}{\beta\:}\right)lnt$$

*α* is the initial fixation rate, and *β* is the sorption constant.

Power function is represented by Eq. (9)

9 $$\:{q}_{t}={\alpha\:t}^{b}$$

*b* is the fixation rate coefficient constant.

Intraparticle diffusion is presented by Eq. (10)

10 $$\:{q}_{t}=k{t}^{1/2}+C$$

*k* is the rate constant of interparticle transport, and C is a constant.

Thereafter, the efficiency of Cr(VI) ions removal (RE%) from aqueous solutions was calculated using Eq. (11).

11 $$\:RE\%=\frac{{C}_{0}-{C}_{t}}{{C}_{0}}\times\:100$$

where *C*_0_ and *C*_t_ are concentrations of Cr(VI) ions at the start of the experiment and each time interval, respectively.

As mentioned by Shariatmadari et al. (2006), the standard error of estimates was calculated as Eq. (13).

12 $$SE = {\rm{ }}[\sum {{{({Q_t} - Q_t^i)}^2}/(n - 2)} ]$$

where ${Q_t}$and ${Q_t^i}$are the measured and predicted concentrations of Cr(VI) ion at time *t*, respectively, and “*n*” is the number of measurements.

### Response surface methodology (RSM)

Using RSM, the optimization of factors influencing the removal of Cr(VI) in the presence of AC600 adsorbent was investigated. Design-Expert version 13.0.5.0 was the program used, and the D-Optimal design (DOD) was applied. For this, the starting Cr(VI) concentration, reaction duration, and adsorbent dose were chosen^28,29,30,31,32,33,34,35,36^ . Table 1 lists the parameters under study along with their corresponding levels. Cr(VI) elimination percentage (%) was the reaction under investigation. Twenty experiments were carried out using various combinations of factors. Table 1 presents the planned experiments.

**Table 1:** The variety of parameters that were examined and employed throughout the optimization process.

Factor	Name	Units	Minimum	Maximum	Mean	Std. Dev.
A	Dose	g/L	0.500	2.50	1.35	0.8288
C	Cr(VI) Conc.	mg/L	100.00	400.00	237.50	133.65
B	Time	Min	15.00	120.00	70.50	44.10

### ANN modelling

Artificial neural network (ANN) modelling uses biological human brain networks to forecast input and output data correlations. There are three primary components of feed-forward back-propagation neural networks (BPNNs), the most popular of which are an input layer (IL) (independent variable), hidden layers (HNs), and an output layer (OL) (dependent variable). MATLAB R2015b version uses the Levenberg-Marquardt (LM) training algorithm to represent the removal of Cr(VI) ion by AC600. The Levenberg-Marquardt (LM) training algorithm consists of training data (70%), validation data (15%), and testing data (15%). The optimal BPNN comprises two hidden layers (HL) of 7 neurons each. Finally, the inputs were the adsorbent dosage of AC600 (g/L), time (min), and Cr(VI) ion initial concentration (mg/L). The removal of Cr(VI) ion was the output variable^37,38,39,40^.

## Results and discussion

### Characterization

#### Analysis of the AC600 morphology

**Fig. 1:**
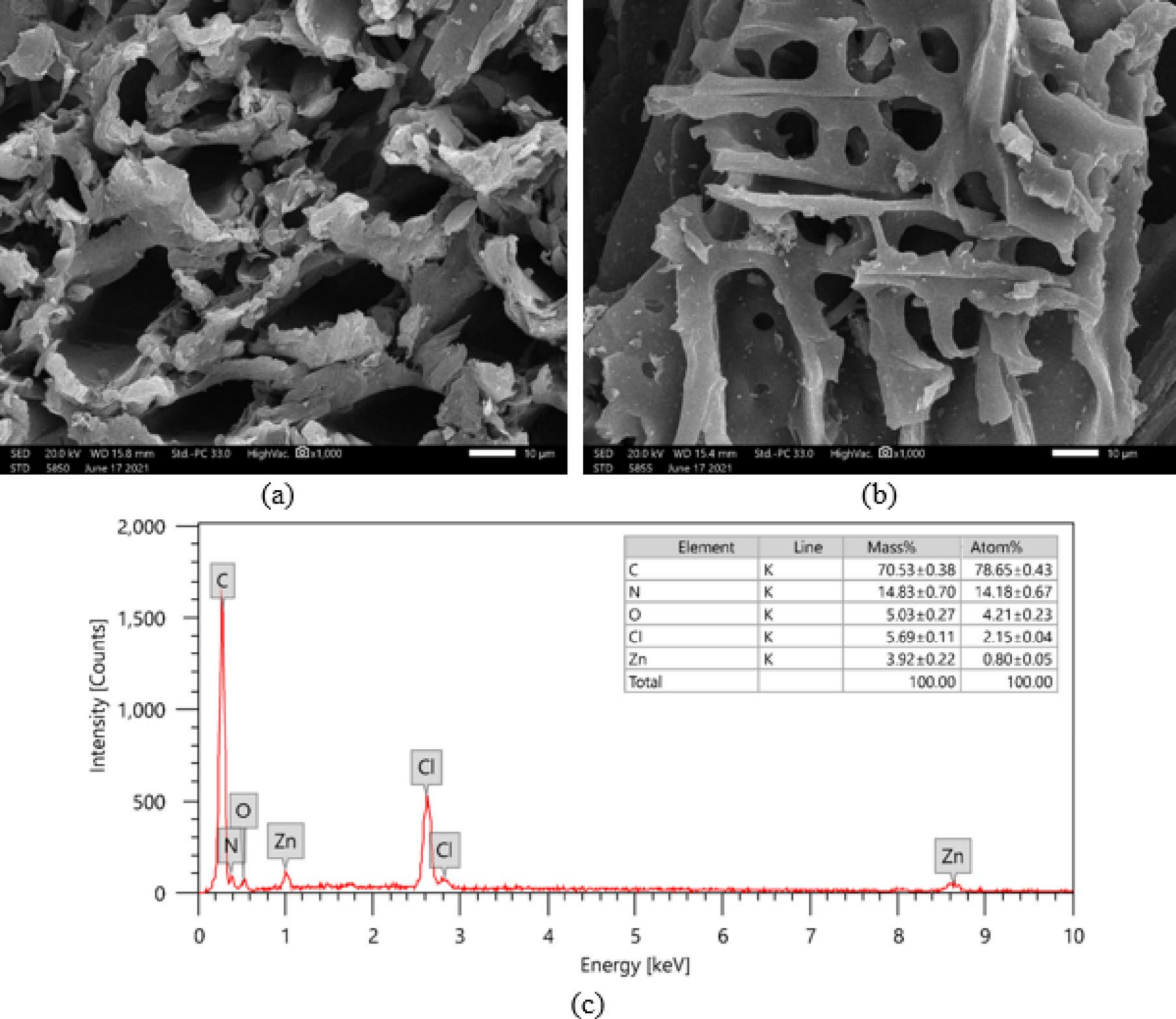
SEM image of (**a**) AC600, (**b**) AC600, (**c**) EDX of AC600.

The surface morphology SEM pictures of the generated AC600 from the SD and ZnCl_2_ combination under NH_3_ gas flow are shown in Fig. 1. Figure 1’s SEM picture showed that the materials’ macroscopic surface was heterogeneous, with many folds, crinkles, and pericarps^41,42^. A rough and smooth SA from the AC600 is visible in the SEM picture in Fig. 1a and b. A few macropores were seen, and these smooth regions were described by a rough structure resembling a series of parallel lines. These rough surface micrographs show a variety of oval-shaped roughness. It was evident from the oval shape that the AC600 had macropores. The AC600 pore size was 20 μm, with an average pore size of 25 ± 5.8 μm based on Fig. 1. Based on Fig. 1c, the EDX of the prepared AC600 shows the elemental composition was 70.53 ± 0.38%, 14.83 ± 0.70%, 5.03 ± 0.27%, 5.69 ± 0.11%, and 3.92 ± 0.22% of carbon (C), nitrogen (N), oxygen (O), chlorine (Cl), and zinc (Zn) elements, respectively (Fig. 1c).

The gas-phase ammonolysis process at 600 °C, which breaks down ammonia into active species like NH_2_-radicals and atomic nitrogen that integrate into carbon vacancies and edges to form stable pyridinic, pyrrolic, and graphitic N configurations, is responsible for the high nitrogen content in AC600 following NH_3_ activation. Because the lower temperature preserves labile N species while etching micropores for better dispersion, this approach surpasses liquid-phase doping (such as urea or melamine treatments) by limiting N loss by volatilization, obtaining 8–12 weight% N as opposed to 2–5 weight% in urea-derived carbons. When compared to other N-doped carbons, like those from urea-impregnated AC (3–6 wt% N) or melamine pyrolysis (4–7 wt% N, lower Cr(VI) capacity ~ 200 mg/g), AC600’s superior retention is attributed to NH_3_’s direct grafting, as demonstrated by XPS peaks at 399.54 eV (pyridinic N) dominating over 400.5 eV in competitors, improving reduction kinetics at pH 1.5.

#### Analysis of the FTIR

**Fig. 2:**
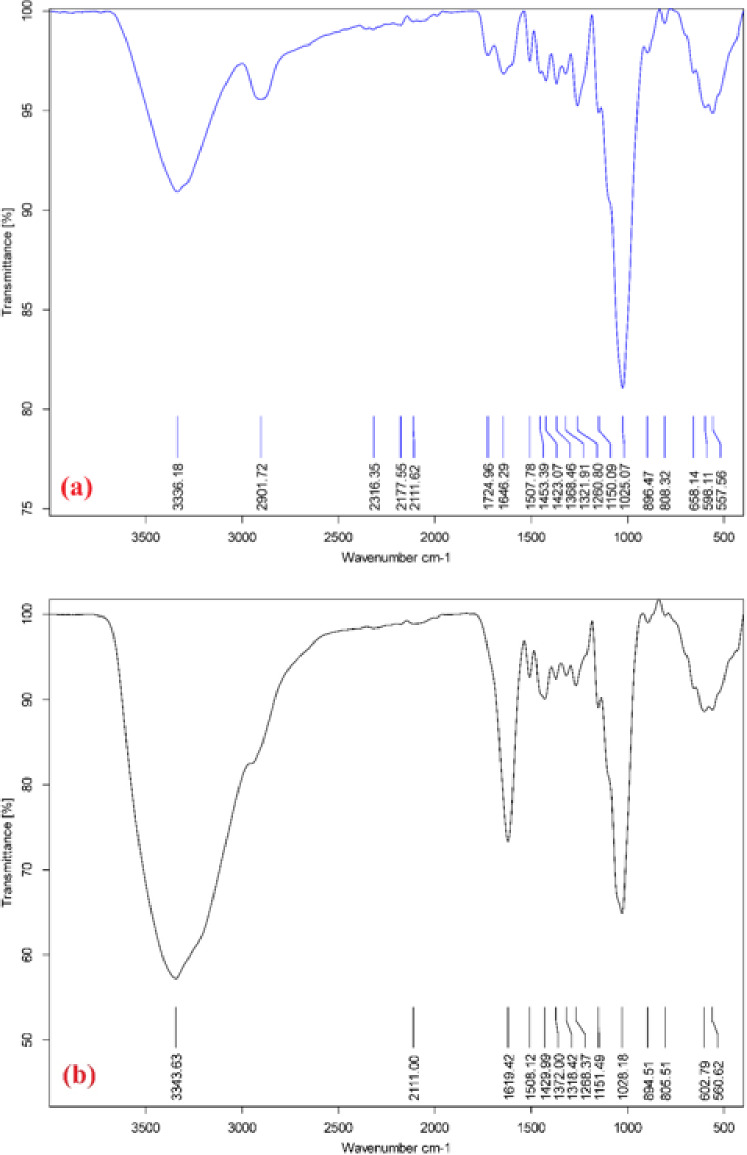

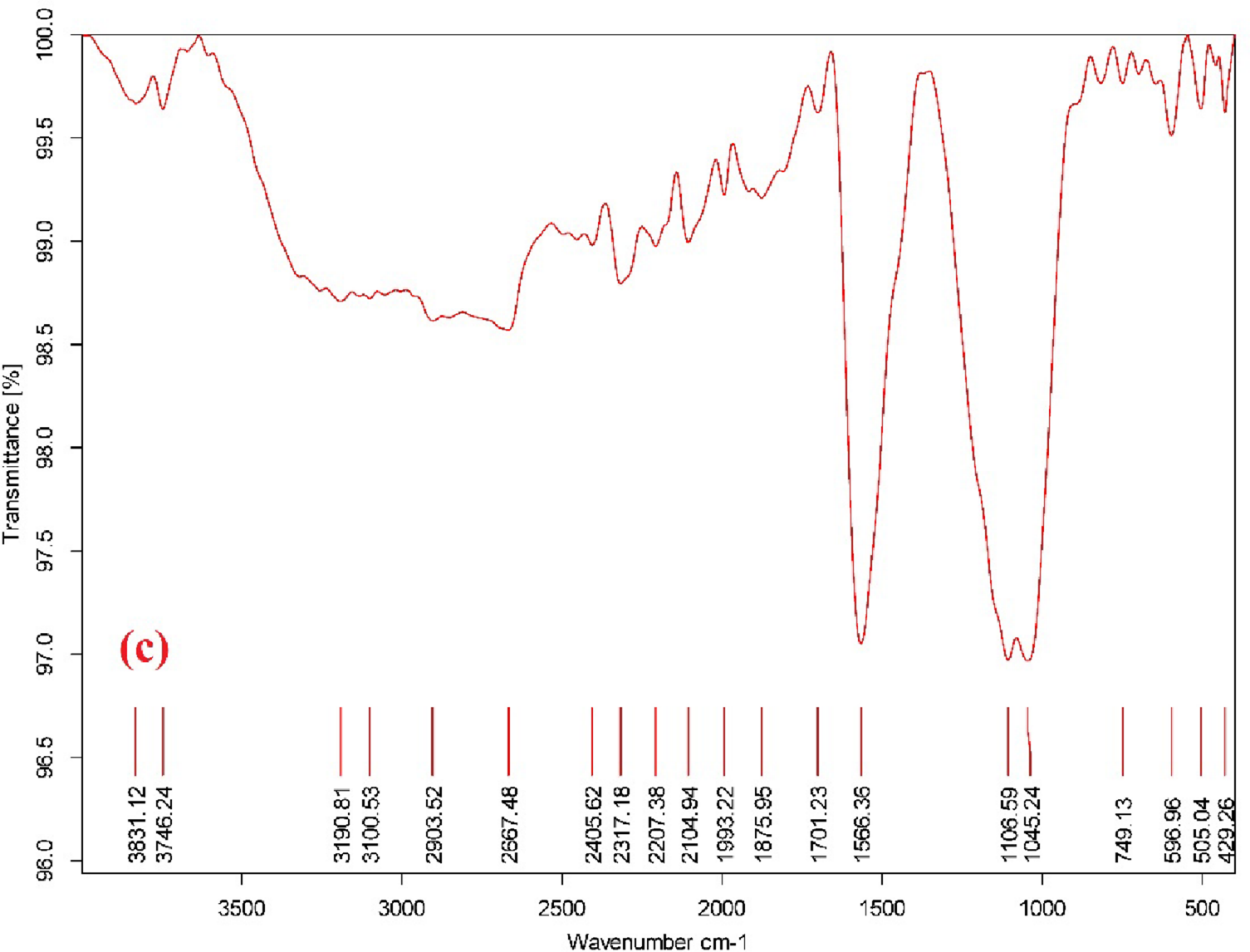
FTIR spectra of (**a**) Saw Dust, (**b**) Saw dust + ZnCl_2_, and (**c**) AC600.

Figure 2a–c shows the FTIR spectra of the raw sawdust, sawdust combination with ZnCl_2_, and the AC600 obtained from the mixture of raw SD and ZnCl_2_ treated with NH_3_ gas at 600 °C. The sawdust FTIR spectra are displayed in Fig. 2a. The presence of intermolecular bound -OH stretching of lignin and cellulose groups, stretching of aliphatic CH_2_ groups, and C-H stretching vibration from –CH_2_ groups were identified as the causes of the adsorption peaks at 3336.18 and 2901.72 cm^–1^. The aldehyde C=O stretching vibration from the aromatic groups of lignin, the N–H amide group, the C=C stretching in the aromatic compounds, and the -OH deformation were attributed to the peaks seen at 1768.46, 1646.29, 1507.78, and 1423.07 cm^–1^. Peaks seen at 1025.07, 896.47, 808.32, 658.14, 598.11, and 557.56 cm^–1^ were ascribed to aromatic compound bending vibration and C–O stretching of the main alcohol. The aromatic skeletal vibration was the cause of the peak at 896.47 and 808.32 cm^–1^^43,44^. Figure 2b displays the ZnCl_2_ and sawdust FTIR spectra. The -OH groups of cellulose were identified as the cause of the broad peak at 3343.63 cm^–1^. A little peak at 2900.51 cm^–1^ was attributed to the sawdust’s CH_2_ stretching vibrations. There were stretching vibrations of C=O: ZnCl_2_ and C=C: ZnCl_2_ bending and stretching vibrations responsible for 1619.42, 1508.12, 1429.99, 1372.00, 1318.42, and 12628.37 cm^–1^. Peaks at 1151.49 and 1028.18 cm^–1^ were caused by vibrations from O-H bending and stretching^45,44^. Meanwhile, Fig. 2c depicts the FTIR spectra of AC600. The peaks at 3831.12 and 3746.24 cm^–1^ represent the N–H stretching frequencies, and the peaks at 3190.81 and 3100.53 cm^–1^ represent the –OH stretching for alcohol frequencies. Meanwhile, the peaks at 2903.52 and 2667.48 cm^–1^ were ascribed to CH_2_ stretching vibrations of CA600. The peaks in the 2405.62–1566.36 cm^–1^ range were ascribed to the C–H vibration in –CH_2_– deformation, the N–H bending and C–N stretching vibration of amide II, and the C–O–C stretching and external bending of –C–H for various substituted benzene rings^18,44^. The ketamine (C=C=N) and C=N stretching vibrations were located at 2100 and 2300 cm^–1^^46^. No, a C=C=N (ketenimine) group at 2100–2300 cm^–1^ is not confirmed by standard FTIR assignments for activated carbon; this peak probably represents misassignment of C–N (nitrile) or C–C stretches from nitrogen doping or impurities, as ketenimines are uncommon in carbons and usually appear ~ 2100 cm^–1^ only in synthetic polymers, not NH_3_-activated AC. The analysis of peak shifts and intensity changes (particularly for N–H/O–H and C–N/C=N groups) provides strong evidence for the coordination and electrostatic adsorption mechanisms of Cr(VI) to the active sites^46^.

#### Analysis of the XRD

**Fig. 3:**
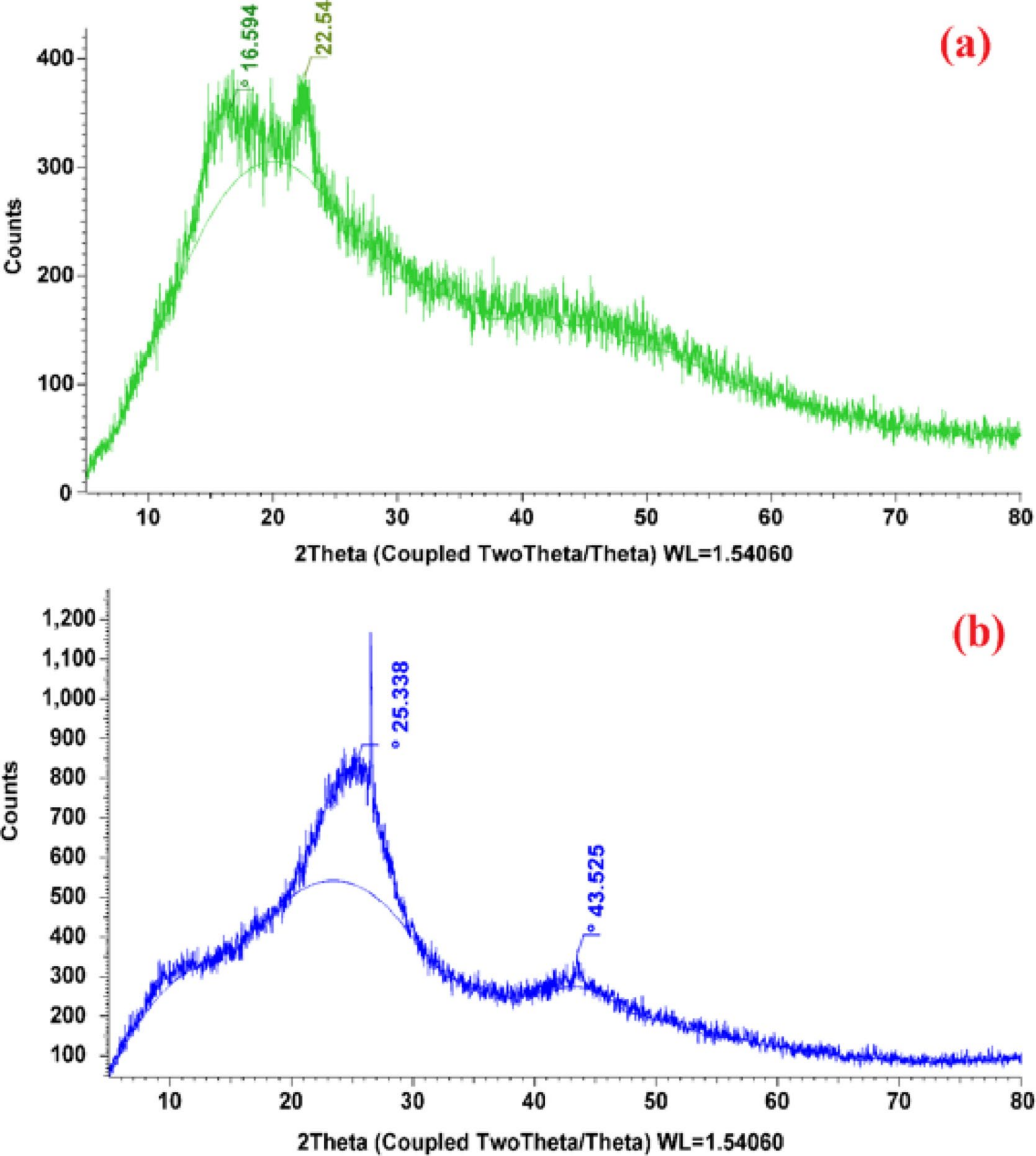
XRD pattern of (**a**) SD and ZnCl_2_ and (**b**) synthesized N-doped AC600.

Cellulose may be crystalline and amorphous in lignocellulosic biomass, but lignin and hemicellulose are considered amorphous. Figure 3a shows the XRD pattern of the ZnCl_2_ and raw sawdust. For the raw biomass, the crystalline plane peaks of raw sawdust were located at narrow, sharp 2θ values of 16.59 and 22.54°; the lower value indicated the material’s amorphous area^47,48,49^. There are crystalline and amorphous sections in the structure, as can be seen in the XRD pattern of raw sawdust and ZnCl_2_, which is comparable to the XRD pattern of cellulose^50,51^. Figure 3b shows the AC600’s XRD spectrum. According to the JCPDS card no. 41-1487, the 002 and 100 interplanar spacing planes of carbon and the planes of hexagonal graphite were responsible for the broad and weak peaks seen at 2θ values of 25.34° and 43.53°. This demonstrated the presence of turbostratic carbon, a low degree of crystallinity (15.4%), and a trivial graphitic crystal structure with a size of a few nanometres in this porous carbon. Because of the activation process, the broad peak (002) shows a characteristic of unstructured carbon, and the peak at 100 indicates that graphitic carbon was present in the sample. By hydrolyzing and dehydrating lignocellulosic components and producing micropores through chemical etching, ZnCl_2_ activation predominantly destroys the crystalline cellulose structure in raw sawdust, transforming sharp peaks at 16.59° and 22.54° (indicative of cellulose Iβ) into a more amorphous profile. The carbon framework is further amorphized by subsequent NH_3_ treatment at 600 °C, resulting in AC600’s broad XRD peaks at 25.34° (002 plane) and 43.53° (100 plane), which are indicative of turbostratic carbon with poor graphitic order and nanoscale domains according to JCPDS card no. 41-1487. For Cr(VI) adsorption/reduction, this transition from crystalline biomass to disordered, porous turbostratic carbon increases surface area and N-doping sites^43,44,52,53^.

#### X-ray photoelectron spectroscopy (XPS)

**Fig. 4:**
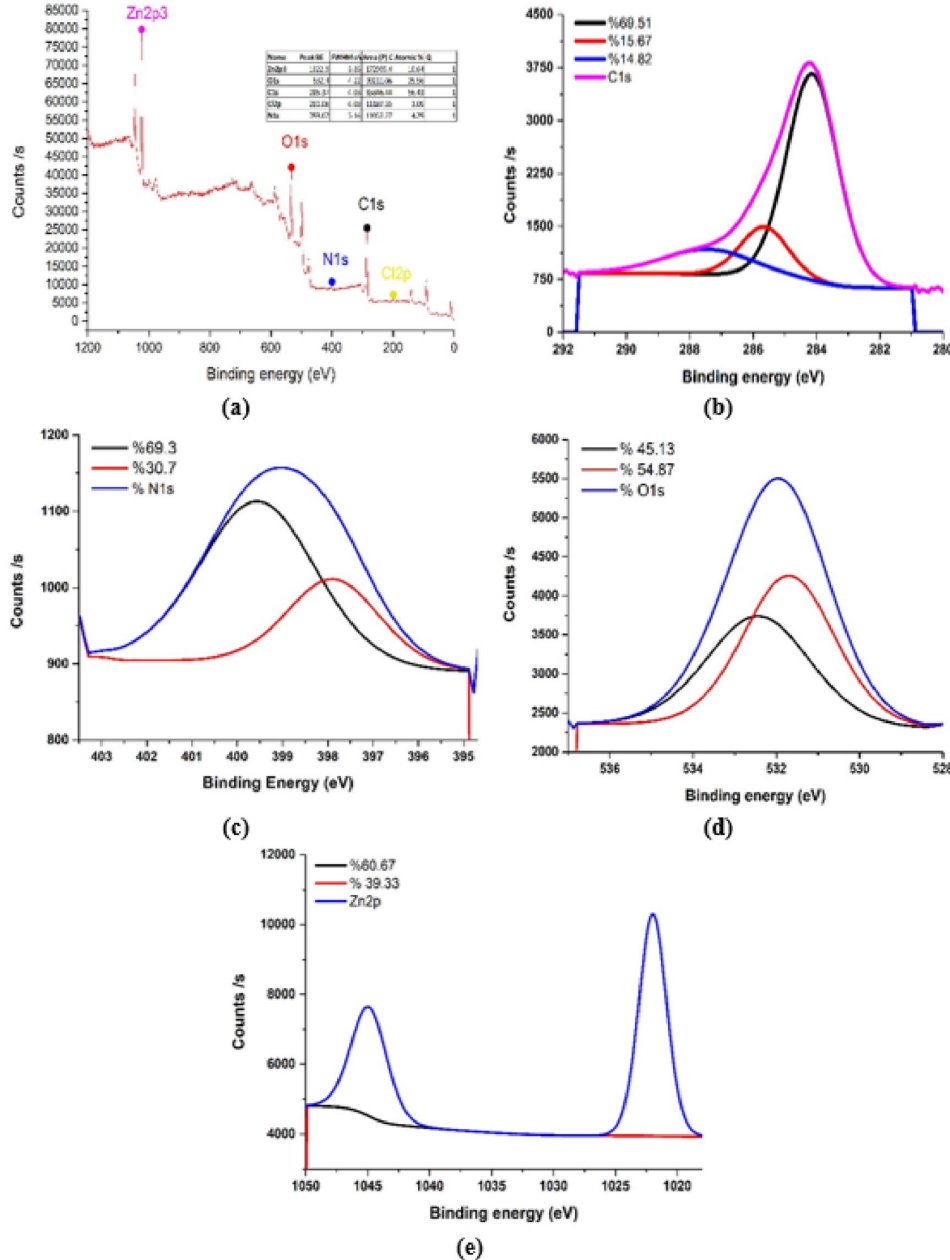
High resolution of XPS core level spectra of (**a**) full scan spectrum, (**b**) C1s, (**c**) N1s, (**d**) O1s and (**e**) Zn2p.

Activated carbon’s surface functional groups were qualitatively examined using XPS^54,55^. The broad complete XPS spectra of the AC600 are shown in Fig. 4a. The figures show that N has been effectively retained on AC600, as seen by the distinctive peaks of C1s, O1s, N1s, and Zn2p. C1s, N1s, O1s, and Zn2p are represented by the peaks at 285.37, 399.67, 532.4, and 1022.9% eV, respectively. Curve fitting of the C1s spectrum shows three peaks in Fig. 4b C1s. Three peaks, centred at 284.14 (69.51%), 285.65 (15.67%), and 287.44 eV (14.82%), can be identified in the C1s spectra. These peaks are attributed to sp2-C hybridized C=C bonds, C−O/C−N bonds, and −O/C=O bonds, respectively^43,56,57^. The N1s XPS spectra of AC600 could be deconvoluted into two types of N-containing compounds, and results are depicted in Fig. 4c. According to Yang et al.^58^, the peaks of N1s in AC600 at 399.54 (pyridinic N) and 397.88 (pyrrolic N) respectively demonstrate that N doping is present in the activated carbon that was generated under the NH_3_ gas condition at 600 °C. The capacitive characteristics are effectively improved by the presence of pyridinic and pyrrolic N, which facilitate ion transport from the electrolyte to the electrode material. The AC600’s O1s XPS spectra shows two peaks at 531.68 eV (45.13%) and 532.76 eV (54.87), which correspond to (C=O) and (C–O), respectively, as seen in Fig. 4d. Figure 4e displays the Zn2p peak of AC600, whereas the binding energies of 1044.99 and 1022.08 eV matched the Zn 2p1/2 and Zn 2p3/2 peaks of ZnO^59^.

#### Analysis of the BET

**Fig. 5:**
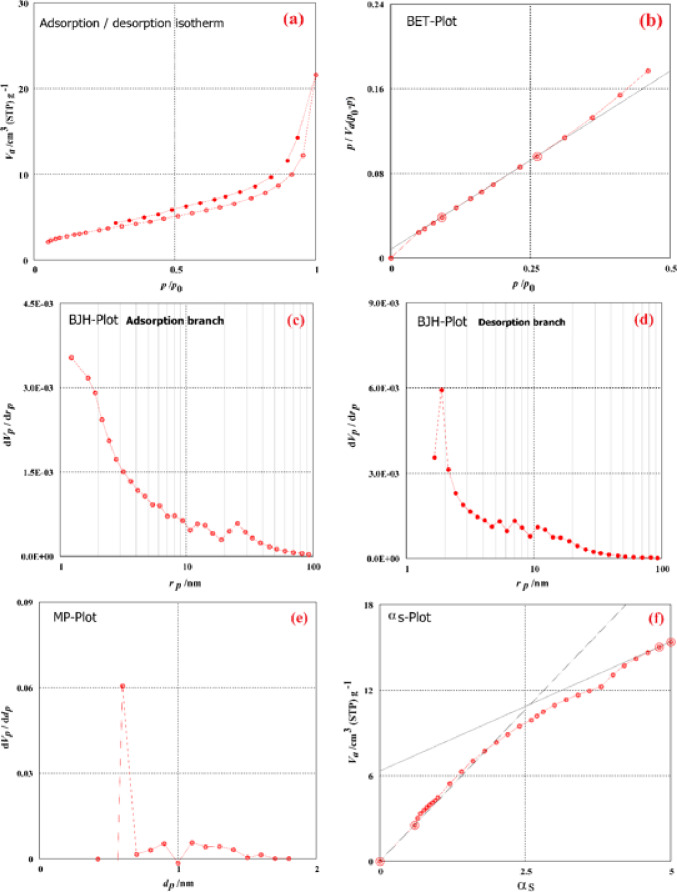
N_2_ adsorption–desorption of N-doped AC600 adsorbent (**a**) Adsorption–desorption, (**b**) BET analysis, (**c**) BJH-adsorption analysis, (**d**) BJH-desorption analysis, (**e**) MP-Plot analysis, and (**f**) α_S_-Plot analysis.

Figure 5 shows the N2 adsorption-desorption isotherm and its various model analyses for the AC600 adsorbent. According to the IUPAC categorization of adsorption isotherms, the AC600’s adsorption isotherm was a typical type V (Mesopore) isotherm because of the highland formed above 0.2 and the sharp rise in the P/Po range of around 0.0-0.2. It displayed a mesoporous and microporous AC substance. The examination of BET data revealed that the AC600 adsorbent’s SA, monolayer volume, mean pore diameter, and total pore volume were 12.622 m^2^/g, 2.9 cm^3^ (STP) g^–1^, 9.57 nm, and 0.0302 m^3^/g, respectively (Table 2). As a result, the derived isotherm profile is affected by the mesoporosity that AC600 displayed^60,61^. The adsorption-desorption isotherm was analyzed using different isotherm analysis models, such as BJH, MP and *α*_S_-plot models and the results were reported in Fig. 5; Table 2.

A low-pressure micropore filling (*P*/*P*_0_ = 0.0–0.2) and a strong mesopore capillary condensation above *P*/*P*_0_ > 0.2 characterize the type V profile of the N2 adsorption–desorption isotherm, as illustrated in Fig. 5. According to BJH, MP, and αs-plot analyses, this hierarchical porosity results in a BET surface area of 12.622 m^3^/g, monolayer volume of 2.9 cm^3^ (STP) g^–1^, total pore volume of 0.0302 m^3^/g, and mean pore diameter of 9.57 nm (Table 2). Adsorption is directly improved by these characteristics: While micropores (by MP and αs-plot) offer high-affinity binding sites that increase capacity by approximately 25% in comparison to non-porous analogs, mesopores (via BJH) enable quick mass transfer and accessibility for pollutants^60^;^61^. N-doping increases selectivity by introducing basic pyrrolic/quaternary sites that encourage π–π interactions and electrostatic attraction for aromatic/heavy metal adsorbates. As demonstrated by improved absorption kinetics in performance testing, residual ZnO nanoparticles function as Lewis acid sites, facilitating chemisorption via coordination with electron-rich contaminants.

**Table 2:** Surface area analysis of AC600 adsorbent.

Method	Parameter	Value	Unit
BET analysis	Monolayer volume (*V*_m_)	2.9000	cm^3^ (STP) g^–1^
	Energy constant (the first layer) (*C*)	42.672	
	Mean Pore diameter	9.5702	nm
	BET specific surface area (*a*_s, BET_)	12.622	m^2^ g^–1^
	Total Pore volume	3.0199 × 10^–2^	cm^3^ g^–1^
BJH analysis adsorption	Pore Volume (*V*_p_)	2.9228 × 10^–2^	cm^3^ g^–1^
	The pore specific surface area (*a*_p_)	10.818	m^2^ g^–1^
	Micropore radius (cylindrical shape) (*r*_p, peak_ (Area))	1.22	nm
BJH analysis desorption	Pore Volume (*V*_p_)	2.8335 × 10^–2^	cm^3^ g^–1^
	The pore specific surface area (*a*_p_)	8.8571	m^2^ g^–1^
	Micropore radius (cylindrical shape) (*r*_p, peak_ (Area))	1.88	nm
MP-Plot analysis	Total specific surface area (*a*_1_)	13.360	m^2^ g^–1^
	External surface area (*a*_2_)	9.6042	m^2^ g^–1^
	Pore volume (*V*_P_)	3.0589 × 10^–3^	cm^3^ g^–1^
	Micropore diameter (*d*_P_)	0.60	nm
*α*_S_ plot analysis	Total specific surface area (*A*_1_)	10.172	m^2^ g^–1^
	External surface area (*A*_2_)	4.3332	m^2^ g^–1^
	Pore volume (*V*_1_)	0.0000	cm^3^ g^–1^
	Pore volume (*V*_2_)	9.7856 × 10^–3^	cm^3^ g^–1^

#### Analysis of the TGA

**Fig. 6:**
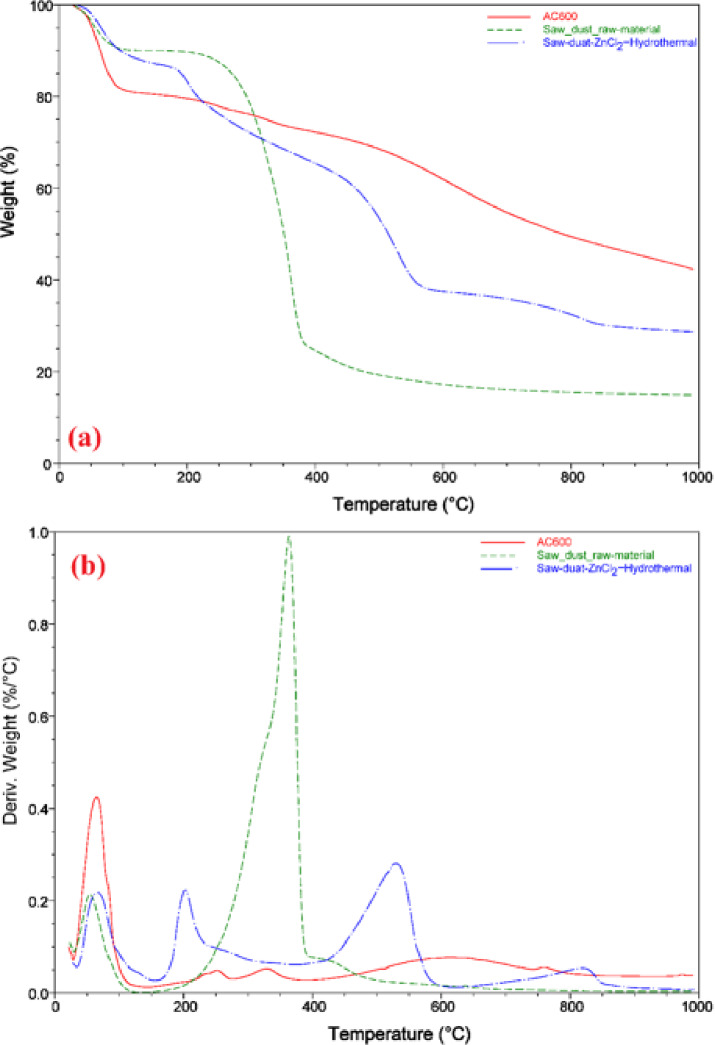

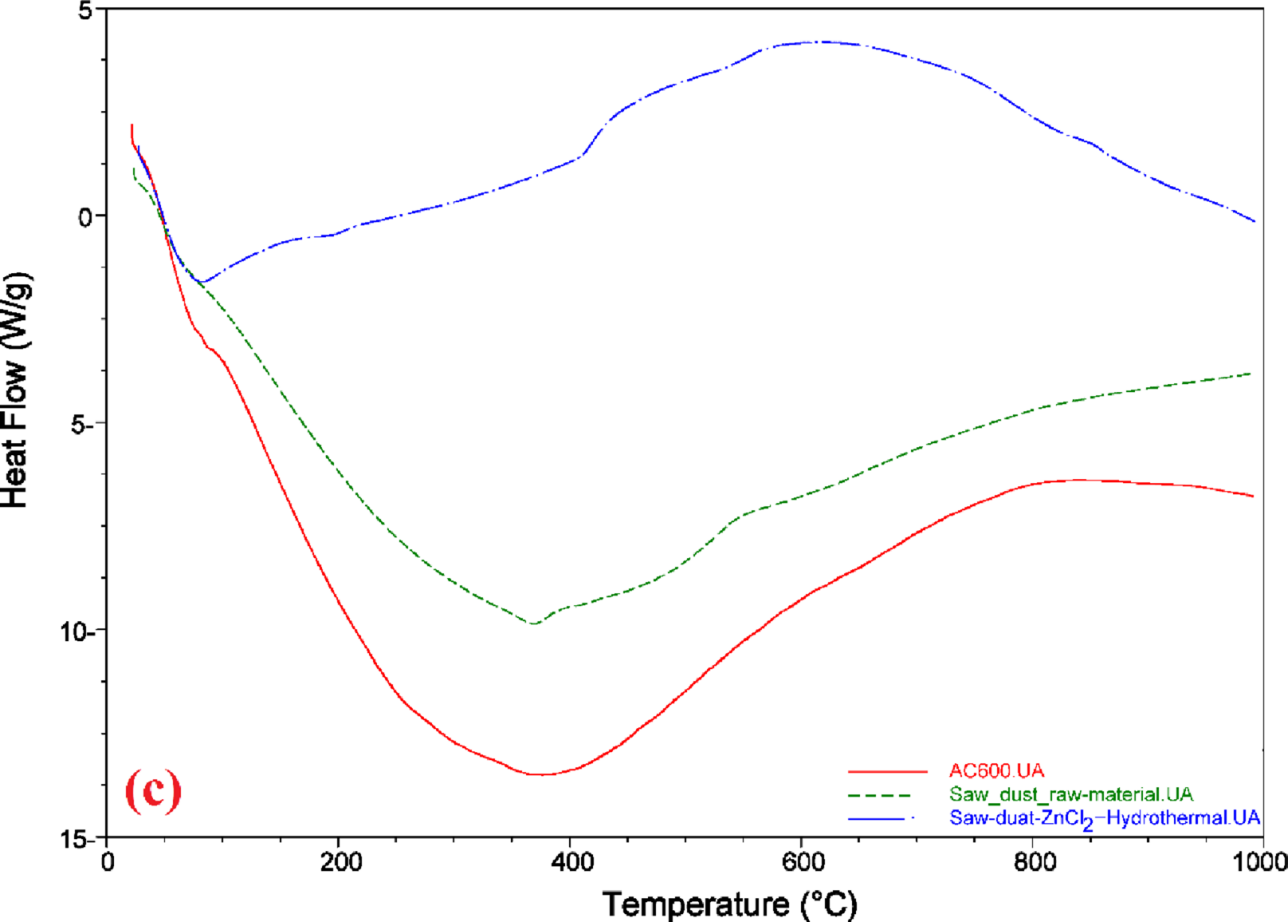
(**a**) TGA, (**b**) DRA, and (**c**) DSC studies of Sawdust, Sawdust-ZnCl_2_-Hydrothermal and AC600.

Figure 6 shows the TGA, DTA and DSC analysis of the Saw dust, Saw dust-ZnCl_2_-Hydrothermal and AC600. Different phases of material degradation are revealed by TGA, which reflects the chemical development from lignocellulosic biomass to N-doped carbon. The 50–200 °C step (9.79% loss) for raw sawdust is associated with moisture evaporation and low-molecular-weight volatiles; the 200–700 °C step (55.12%) is associated with the pyrolysis of hemicellulose (200–300 °C), cellulose (300–400 °C), and lignin (400–700 °C) through depolymerization and charring; and the 700–1000 °C (22.71%) is associated with char oxidation or additional graphitization. Sawdust-ZnCl_2_-Hydrothermal exhibits shifted phases: 50–150 °C (12.98%) for bound water/Zn complexes; 150–350 °C (20.10%) for hemicellulose breakdown facilitated by ZnCl_2_ catalysis; 350–620 °C (29.63%) for cellulose/lignin dehydration; and 620 °C (8.60%) for ZnCl_2_ volatilization, which leaves etched carbon residue. High thermal stability after NH_3_ activation is confirmed by AC600’s simplified carbon-dominated losses, which are 50–150 °C (19.33%) for residual moisture/nitrogen volatiles, 150–260 °C (3.73%) for labile N-groups (such as amines), 260–400 °C (4.59%) for C-O/C-N decomposition, and 400–1000 °C (29.90%) for turbostratic carbon burnout (Fig. 6a).

Figure 6b shows the DTA curve peak of the Sawdust, Sawdust-ZnCl_2_-Hydrothermal and AC600. The DTA curve of the raw Saw dust peaked at three temperature points (*T*_*f*_, 92.36, 443.80 and 790.00 °C), and the curve of Saw dust-ZnCl_2_-Hydrothermal peaked at four temperature points (*T*_*f*_, 67.03, 202.18, 530.60 and 819.77 °C) (Fig. 6b). The DTA curve of the AC600 peaked at five temperature points (*T*_*f*_, 65.52, 251.27, 329.33, 610.18 and 761.44 °C) (Fig. 6b) The DTA curve demonstrating the production of AC600 adsorbents from Sawdust-ZnCl_2_-Hydrothermal indicates that the dehydration of Sawdust-ZnCl_2_-Hydrothermal yielded five distinct degradation bands. The Saw dust-ZnCl_2_-Hydrothermal degradation bands occurred at a higher temperature, demonstrating that the degradation degree was intensely affected by treatment at 600 °C.

Glass transitions or thermal transitions can be utilized in DSC to compare materials. Figure 6c depicts the DSC graph of the Sawdust, Sawdust-ZnCl_2_-Hydrothermal and AC600. The glass transition temperatures (*T*_g_) of Sawdust occurred at three sets (113.78, 125.93, and 148.28), (305.32, 371.97, and 475.29), and (674.79, 695.19, and 706.39) °C. The glass transition temperatures (*T*_g_) of Sawdust-ZnCl_2_-Hydrothermal at two sets (37.21, 49.00, and 62.23), and (422.42, 434.75, and 498.60) °C. The glass transition temperatures (*T*_g_) of AC600 at two sets (40.80, 131.96, and 238.20 °C, and 443.26, 500.95, and 730.30 °C). While the Saw dust, Saw dust-ZnCl2-Hydrothermal and AC600 display onset point values at (116.52, 476.25, and 706.25) °C, (62.24 and 506.54) °C, and (146.70 and 730.94) °C. Since genuine “glass transition” (Tg) refers to amorphous polymers where chain mobility shifts rather than rigid lignocellulosic biomass or turbostratic carbons like AC600, which do not exhibit such viscoelastic behavior, the DSC transitions designated as “glass transition” (Tg) for these materials are scientifically unsuitable. Rather, XRD’s shift from cellulose peaks (16.5°, 24.1°) to broad turbostratic (002/100) planes confirms that these multiple “Tg” peaks (e.g., Sawdust at 113–706 °C, Sawdust-ZnCl_2_-Hydrothermal at 37–498 °C, AC600 at 40–730 °C) represent endothermic events from hemicellulose/lignin devolatilization, ZnCl_2_-catalyzed dehydration, and carbon framework reorganization. Higher temperatures after activation imply improved crystallinity and resistance to breakdown rather than glass transitions; reinterpreting these as “thermal transition events” is consistent with normal carbon pyrolysis research. Onset points (116–730 °C) show thermal stability limits.

### AC6-600 adsorption isotherms for chromium removal

**Fig. 7:**
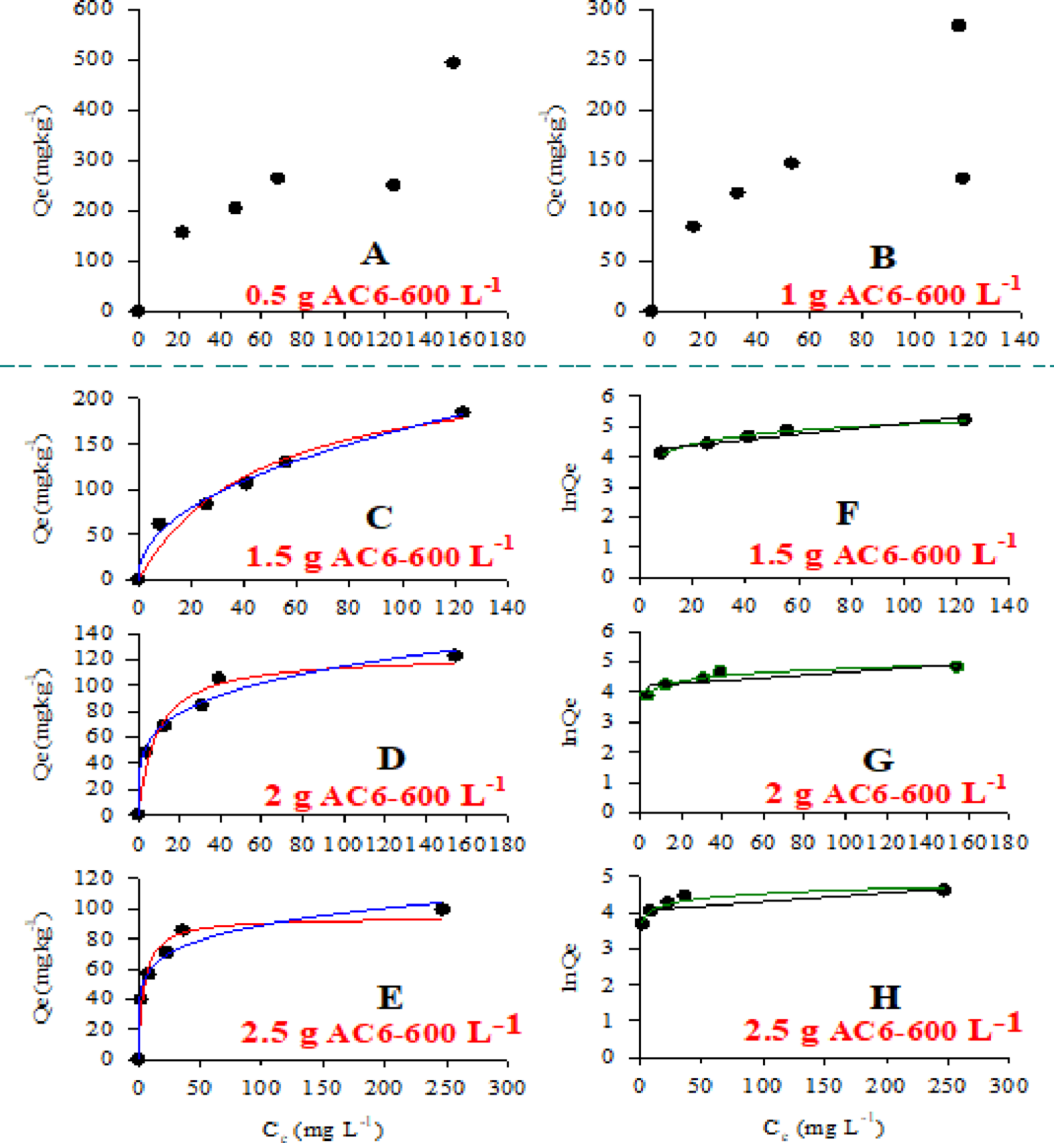
Adsorption isotherm models of Cr(VI) ions on AC600 across different applied dosages (0.5–2.5 g/L): red (Langmuir isotherm fittings), blue (Freudlich isotherm fittings), black (Temkin isotherm fittings) and dark green (HIM isotherm fittings).

Curves characterizing Cr(VI) ions sorption on either 0.5–1.0 g AC600 L^–1^ seemed to follow both linear and adsorption isotherm models (Fig. 7). At lower doses (< 1.5 g/L), the linear model likely contributed considerably (physical partitioning within the C-matrix), making classical saturation isotherm models less suitable. However, hyperbolic adsorption (indicating adsorption) modelling became more noticeable at doses ≥ 1.5 g/L of AC600. To test the dominant Cr(VI) ions sorption mechanism on activated carbon, sorption data of Cr(VI) ions at doses ≥ 1.5 g/L of AC600 were fitted to Langmuir, Freundlich, Hasley and Temkin isotherm models, and the calculated parameters are presented in Table 3.

Results revealed that Cr(VI) ions sorption onto AC600 surfaces followed the Freundlich isotherm model, as evidenced by higher r^2^ values across all tested AC600 doses. This can therefore be characterized by an adsorption process on heterogeneous sites in multilayers. Higher AC600 doses raised both (1) total sorption capacity due to greater availability of binding sites towards Cr(VI) ions and also (2) the Freundlich exponent (*n*) values, which increased from 2.188 to 5.848.

A point to note is that the R^2^ values of Halsey Isotherm (HIM) were low, indicating that the sorbed Cr(VI) ions did not exist in multilayers at relatively large distances from the surface^62^, but instead, Cr(VI) ion sorption may be interacting relatively close to the surface of the activated carbon. The interactions of Cr(VI) ions on sorbed surfaces are almost spontaneous and endothermic^63,64^, thus it is no wonder that the slope of the Temkin model—reflecting the sorption energy per unit mass—decreased with increasing initial Cr(VI) ions concentration in the media. These reductions in sorption energy were mostly attributed to site saturation on the activated carbon surface, corresponding to weaker adsorbate–adsorbent interactions^65,66^.

**Table 3:** The determined parameters for the Cr(VI) ion adsorption isotherm models on activated carbon at various applied doses (0.5–2.5 g/L).

AC600 dose	Langmuir isotherm		Freundlich isotherm		Halsey isotherm		Temkin isotherm	
1.5 g/L	*R* ^2^	0.960	*R* ^2^	0.993	*R* ^2^	0.905	*R* ^2^	0.970
	*Q*_max_	245.44	*Q*_max_	20.25	*B*	132.78	Ln A	7.752
	b	0.0215	*n*	2.188	lnA	0.009	*B*	0.411
2.0 g/L	*R* ^2^	0.964	*R* ^2^	0.976	*R* ^2^	0.609	*R* ^2^	0.954
	*Q*_max_	123.54	*Q*_max_	38.687	*B*	834.2	Ln A	13.723
	b	0.115	*n*	4.237	lnA	0.005	*B*	0.260
2.5 g/L	*R* ^2^	0.959	*R* ^2^	0.972	*R* ^2^	0.488	*R* ^2^	0.969
	*Q*_max_	94.33	*Q*_max_	40.447	*B*	1026.5	Ln A	18.030
	b	0.225	*n*	5.848	lnA	0.002	*B*	0.200

According to the Langmuir model, adsorbate-adsorbent interactions are absent and monolayer adsorption occurs on a homogeneous surface with fixed binding sites. Lower *R*^2^ values (relative to Freundlich) across doses suggest that this mechanism is not prominent, most likely because of surface heterogeneity on AC600. It was examined to see whether Cr(VI) sorption occurred by uniform site saturation. With the highest *R*^2^ values across all tested doses, the Freundlich model provided the best description of the data. In line with the porous structure of AC600, this facilitates multilayer adsorption on heterogeneous sites. The Freundlich exponent and total sorption capacity both increased with increasing doses, indicating positive, cooperative adsorption. Multilayer adsorption at great distances from the surface was ruled out by the Halsey isotherm model (HIM), which produced low *R*^2^ values. Rather, intimate interactions between Cr(VI) ions and AC600 sites are consistent with short-range forces and physical partitioning. According to the Temkin model, interactions between the adsorbent and the adsorbate cause the adsorption heat to decrease linearly. As the initial Cr(VI) concentration increased, its slope dropped, indicating site saturation and weaker interactions at higher coverage^65,66^. This suggests endothermic, nearly spontaneous reactions^63,64^.

Our findings showed that Cr(VI) adsorption onto AC600 follows the Freundlich isotherm, which are consistent with the findings of Schneider et al.^64^ and Duranoğlu et al.^65^, while differing from other studies that reported a better fit to the Langmuir model (Table 4).

**Table 4:** Cr(VI) adsorption isotherm models Reported in previous studies.

Study	Adsorbent used	Best-fitting isotherm model	Notes/key findings
Schneider et al.^64^	Activated carbon produced from coconut shell	Freundlich	Cr(VI) sorption showed multilayer, heterogeneous surface adsorption
Duranoğlu et al.^65^	Activated carbon created from acrylonitrile-divinylbenzene copolymer	Freundlich	Cr(VI) sorption took place on heterogeneous adsorption sites
Gueye et al.^67^	Activated carbon derived from jatropha wood and peanut shells	Langmuir	Monolayer adsorption was the dominant mechanism for Cr(VI) sorption
Kumae and Jena 2017	Activated carbon prepared from fox nutshell	Langmuir	Adsorption capacity increased, supporting monolayer chemisorption behavior
Maneechakr and Karnjanakom^68^	Activated carbons produced from *Combretum quadrangulare* Kurz	Langmuir	Cr(VI) adsorption favored Langmuir due to homogeneous surface assumptions

### Sorption kinetics of Cr(VI) ions on AC600 surfaces

**Fig. 8:**
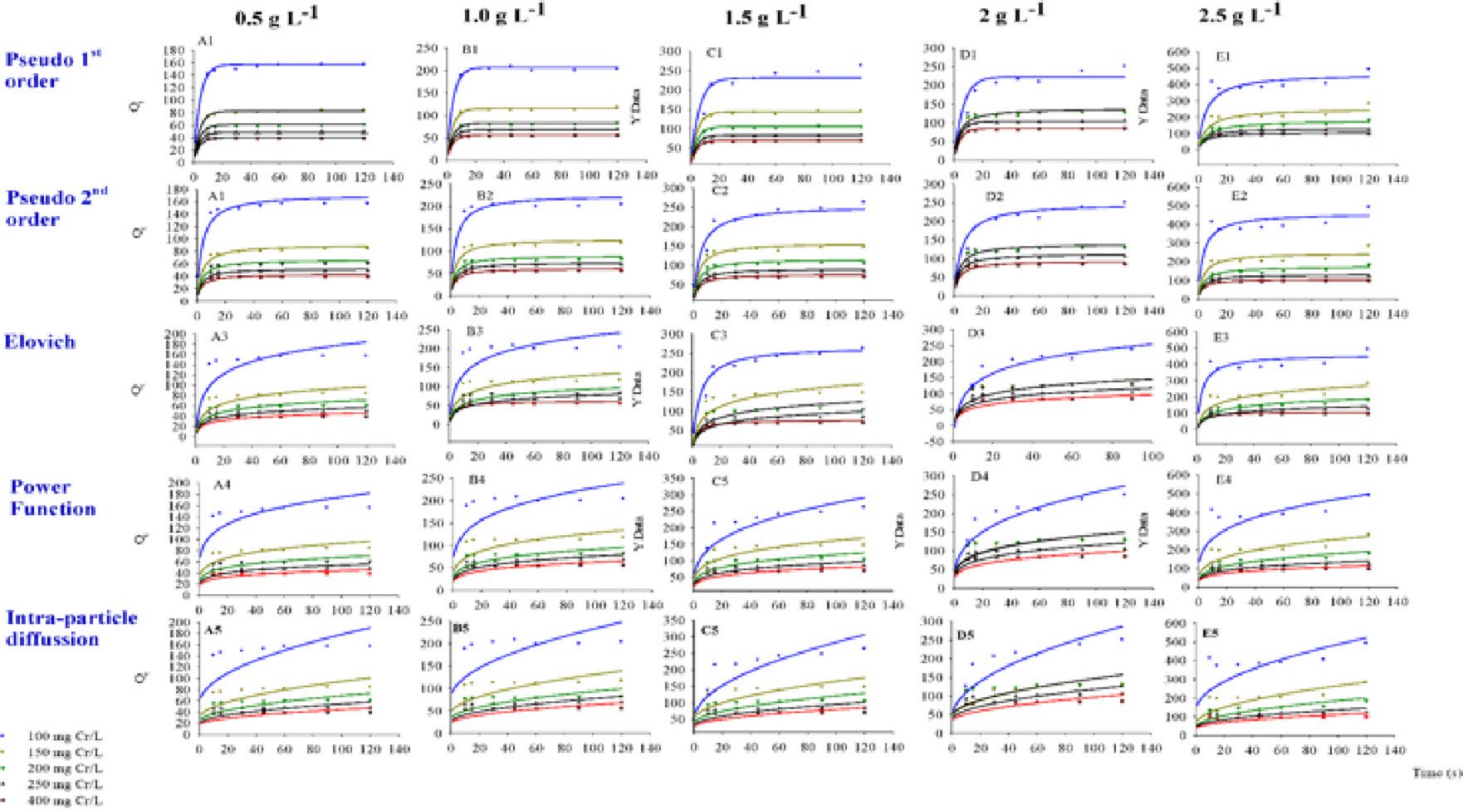
Kinetic modeling of Cr(VI) sorption onto AC600 surfaces across various initial chromium concentrations (100–250 mg/L) and different AC600 dosages (0.5–2.5 g/L).

Sorption kinetics of Cr(VI) ion onto activated carbon, comprising different AC600 dosages (0.5–2.5 g/1000 mL) and Cr(VI) ion concentration (100–250 mg L^–1^), were then evaluated using four kinetic models: Pseudo-First-Order, Pseudo-Second-Order, Elovich, and Power Function models (Fig. 8). The calculated fitting parameters are summarized in Table 5.

The Pseudo-First-Order model generally exhibited low to moderate coefficient of determination (*R*^2^) values ranging from 0.37 to 0.86 while calculated high standard error of estimate (SEE), indicating that this model is not appropriate for describing Cr(VI) ion sorption kinetics in this study, or in other words physisorption is not the rate-limiting step^27,69^ for Cr(VI) ions sorption on AC6-600.

In contrast, the Pseudo-Second-Order model provided the overall best fit, especially at low to moderate dosages of AC600 and at low Cr(VI) ions concentration, with *R*^2^ values ranging from 0.89 to 0.98. This model assumes that Cr(VI) ion removal took place mainly by chemical binding^70^ on the functional groups of AC600, mainly via the surface oxygen functional groups such as carboxylic groups^71^. Likewise, the Elovich model demonstrated good fitting performance (*R*^2^ = 0.90–0.98), particularly within the mid-range Cr(VI) ion concentration. This also supports chemisorption on heterogeneous surfaces^72^.

Surprisingly, the Power Function and intraparticle diffusion models recorded high *R*^2^ values at higher Cr(VI) ion concentrations and AC600 dosages. The intraparticle diffusion model suggests that the rate-limiting step in contaminant sorption from aqueous solutions (Kavranli 2011) is proceeded through several sequential stages, including solute transport in the bulk solution, external or boundary-layer diffusion, internal or intra-particle diffusion, and finally adsorption or desorption onto the interior active sites^73,74^. Results obtained herein probably indicate that Cr(VI) ion removal was proceeded through a two-step process: a fast initial step within macro and mesopores of AC600 and a slower step, where it diffuses deeper inside micro pores of the activated carbon^66^. Therefore, the power function model could fit well when pore diffusion controls the sorption process. Weak physical interactions occurred between AC600 and Cr(VI) ion during these two stages, yet these were not primary mechanisms. Overall, the kinetic analysis indicates that chemisorption is the dominant mechanism for Cr(VI) ion removal by AC600, especially at lower to mid-range Cr(VI) ion concentrations. However, intraparticle diffusion and physisorption processes may contribute at higher adsorbent dosages, highlighting the complexity of the sorption behavior. Our result supports the findings of many other researchers^65,75^ (Yan et al., 2015; Maneecharkr and Karnjanakom, 2017). Doke and Khan^76^ added that the intra-particle pore-diffusion mechanism, as well as pseudo-second order chemisorptions, fitted well Cr(VI) ions sorption on nitrogen-doping activated carbon (AC600) surfaces.

Remarkably, at higher Cr(VI) ion concentrations (≥ 150 mg L^–1^) and AC600 dosages (≥ 1.5 g/L), the Power Function and intraparticle diffusion models reported strong *R*^2^ values (0.92–0.99). Rapid external mass transfer and site saturation in macropores and mesopores are followed by slower diffusion into micropores when the boundary layer thickens, according to the intraparticle diffusion model (Kavranli, 2011)^73,74^. This is consistent with results for Cr(VI) on activated carbons^76,66^ and explains the two-step sorption kinetics shown here—a fast initial chemisorption phase and a diffusion-limited phase at high loading.

**Table 5:** The calculated parameters for Cr(VI) ions adsorption on AC600 across various initial Cr(VI) ion concentrations (100–250 mg/L) and different AC600 dosages (0.5–2.5 g/L).

100 mg L^–1^					150 mg L^–1^				200 mg L^–1^				250 mg L^–1^			
Pseudo 1st order model																
AC600	*R* ^2^	SEE	a	b	*R* ^2^	SEE	a	b	*R* ^2^	SEE	a	b	*R* ^2^	SEE	a	b
0.5 g L^–1^	0.734	0.298	38.886	0.344	0.760	0.771	55.684	0.276	0.804	1.060	69.345	0.255	0.716	1.481	83.687	0.263
1.0 g L^–1^	0.732	0.566	47.448	0.301	0.649	1.226	67.093	0.274	0.660	1.746	82.405	0.256	0.773	2.257	101.887	2.257
1.5 g L^–1^	0.687	1.081	59.752	0.268	0.575	1.255	80.919	0.308	0.744	2.463	104.452	0.230	0.372	6.872	125.181	0.229
2.0 g L^–1^	0.629	2.520	81.349	0.225	0.546	2.191	113.196	0.295	0.773	3.995	142.315	0.205	0.554	3.662	126.120	0.248
2.5 g L^–1^	0.721	3.570	154.016	0.236	0.770	3.348	203.017	0.256	0.439	38.316	230.419	0.110	0.863	16.500	229.499	0.088
Pseudo 2nd order model																
0.5 g L^–1^	0.929	0.154	99.299	2.531	0.968	0.283	70.612	1.247	0.983	0.439	71.975	1.017	0.954	0.594	91.020	1.066
1.0 g L^–1^	0.889	0.364	78.624	1.635	0.927	0.558	80.020	1.171	0.925	0.822	82.663	0.982	0.933	1.227	78.058	0.746
1.5 g L^–1^	0.916	0.560	68.271	1.121	0.758	0.951	141.053	1.721	0.891	1.607	81.948	0.764	0.809	2.201	109.830	0.849
2.0 g L^–1^	0.906	1.271	57.204	0.682	0.604	2.046	182.407	1.590	0.865	3.083	87.824	0.598	0.526	5.968	84.625	0.654
2.5 g L^–1^	0.932	1.766	125.763	0.796	0.644	4.167	263.235	1.278	0.853	17.213	37.982	0.139	0.901	13.997	30.592	0.118
Elovich																
0.5 g L^–1^	0.910	0.174	36.66	0.550	0.957	0.3311	49.374	1.552	0.952	0.523	59.819	2.333	0.975	0.437	72.562	2.741
1.0 g L^–1^	0.903	0.340	43.239	1.039	0.983	0.278	58.814	2.051	0.956	0.630	70.534	2.926	0.949	1.071	83.028	4.615
1.5 g L^–1^	0.965	0.361	52.072	1.897	0.830	0.794	73.864	1.753	0.885	1.649	85.727	4.577	0.935	1.395	104.784	5.299
2.0 g L^–1^	0.987	0.4719	64.691	4.109	0.657	1.905	102.519	2.634	0.842	3.331	110.622	7.686	0.656	5.084	97.093	7.020
2.5 g L^–1^	0.934	1.741	127.410	6.525	0.4335	5.256	183.437	4.596	0.805	19.766	74.230	40.238	0.876	15.686	51.420	41.706
Power function																
0.5 g L^–1^	0.929	1. 800	129.102	0.043	0.428	5.283	184.254	0.023	0.778	21.141	113.543	0.180	0.851	17.2034	95.427	0.204
1.0 g L^–1^	0.987	0.476	64.882	0.052	0.660	1.902	102.858	0.024	0.837	3.386	113.149	0.055	0.6630	5.032	99.102	0.058
1.5 g L^–1^	0.964	0.365	52.425	0.032	0.831	0.792	74.079	0.022	0.881	1.679	86.950	0.045	0.936	1.389	106.053	0.043
2.0 g L^–1^	0.902	0.342	43.376	0.022	0.982	0.276	59.184	0.031	0.954	0.646	71.157	0.036	0.946	1.100	84.273	0.046
2.5 g L^–1^	0.908	0.175	36.712	0.014	0.953	0.343	49.642	0.028	0.949	0.541	60.301	0.034	0.973	0.459	73.104	0.033
Intra-particle diffusion																
0.5 g L^–1^	0.855	2.572	137.750	1.997	0.558	5.793	191.803	1.247	0.722	23.639	138.856	12.186	0.8062	19.617	117.306	12.793
1.0 g L^–1^	0.957	0.857	70.956	1.294	0.824	1.844	106.353	0.856	0.737	3.932	122.714	2.365	0.7002	4.748	107.060	2.319
1.5 g L^–1^	0.930	0.512	54.978	0.595	0.834	0.784	76.469	0.562	0.817	2.084	92.946	1.406	0.912	1.623	112.829	1.674
2.0 g L^–1^	0.855	0.416	44.849	0.323	0.942	0.501	61.969	0.642	0.900	0.948	75.088	0.908	0.898	1.513	90.184	1.436
2.5 g L^–1^	0.839	0.231	37.531	0.169	0.878	0.554	51.830	0.476	0.881	0.827	63.494	0.717	0.86	0.816	76.841	0.848

### Removal efficiencies of Cr(VI) ions

**Fig. 9:**
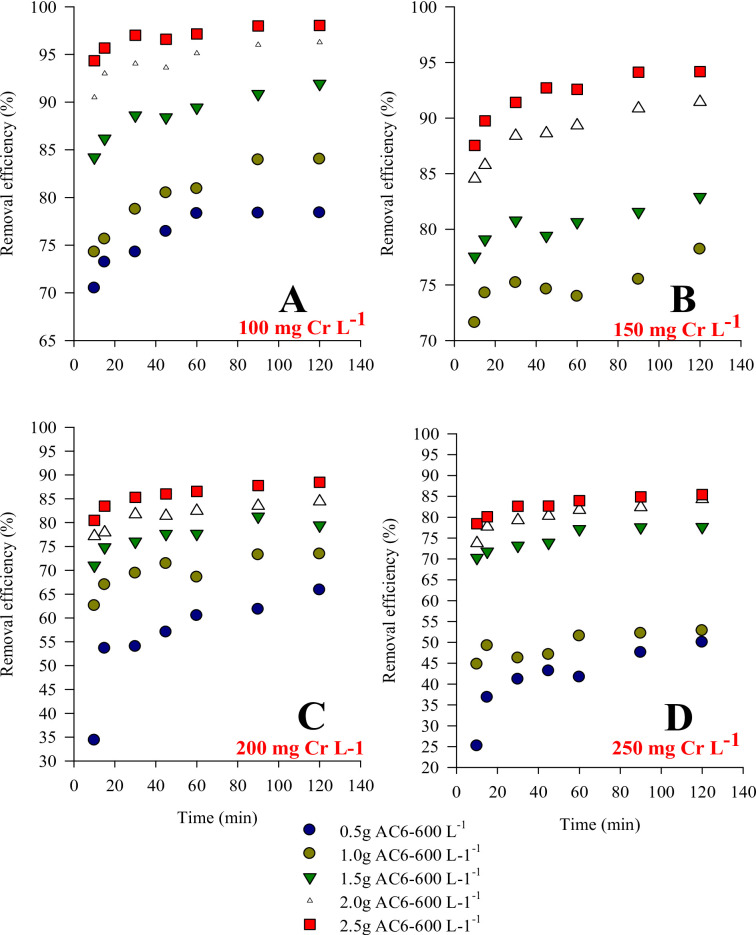
The removal efficiency of Cr(VI) ions from water using AC600 as affected by the initial concentrations of Cr(VI) ions (100–250 mg/L) and different AC600 dosages (0.5–2.5 g/L).

The efficiency of Cr(VI) ions removal from contaminated waters using AC600 increased significantly with increasing the time of contact (Fig. 9). This efficiency was generally influenced by two factors: (1) the dose of applied AC600 and (2) the initial concentrations of Cr(VI) ions in contaminated water. It was noticed that a rapid rise in these efficiencies occurred within the first 20–40 min of contact, indicating fast initial adsorption, then these curves tended to equilibrate within 60–80 min. The lower AC600 doses (e.g., 0.5 g/L) showed significantly lower Cr(VI) ions removal efficiency, which was about 70% for 100 mg Cr L^–1^, and this efficiency decreased to only 35–45% for decontaminating water containing 200–250 mg Cr L^–1^. On the other hand, this efficiency rose considerably up to 95–99% when using 2.5 g/L of AC600 for the removal of Cr(VI) ions from a liter of water contaminated with either 100 or 150 mg Cr L^–1^, while yielding about 88–90% removal for 200 and 250 mg L^–1^ within 120 min of contact. This supports the use of AC600 as an effective adsorbent for Cr(VI) ions, with dose and initial Cr(VI) ions concentration being critical factors. The adsorption of Cr(VI) ions on AC600 was a pH-dependent phenomenon; thus, the removal efficiency of Cr(VI) ions under different pH values was a matter of concern in this study.

### Removal efficiencies of Cr(VI) ions from water as affected by the initial pH of the media

**Fig. 10:**
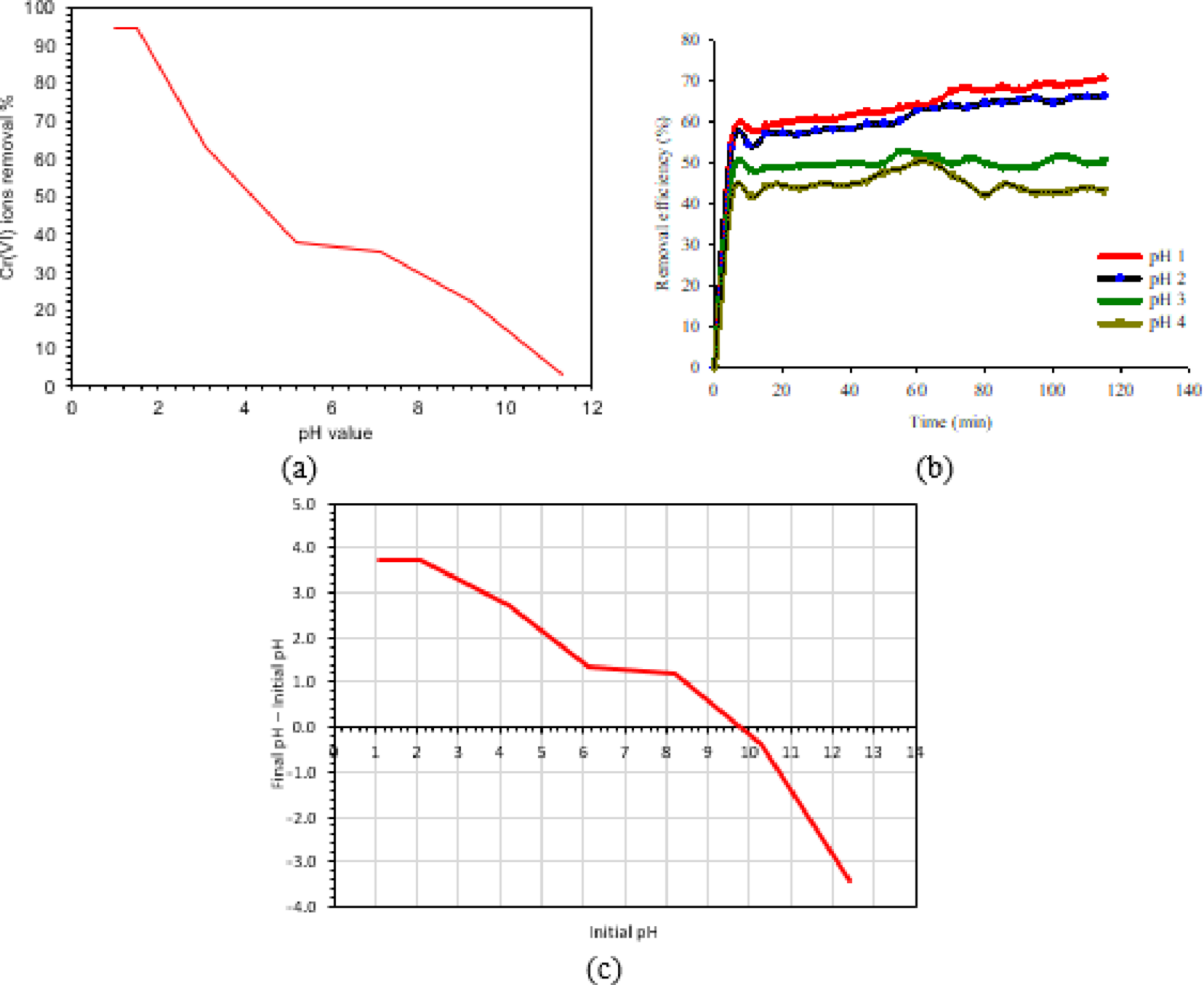
Removal efficiencies of the pH effect on the sorption of Cr(VI) ions (**a**) at pH values 1–12, (**b**) at pH values 1–4 from contaminated water using AC600, (**c**) pH_PZC_ of AC600.

To investigate the effect of pH on the removal efficiency of Cr(VI) ions by AC600, a fixed adsorbent dose with two concentrations of Cr(VI) (100 mg L^–1^ at pH values from 1 to 12, and 200 mg L^–1^ at pH values 1–4) was applied (Fig. 10a,b). Figure 10a shows that the best pH value is pH 1 or 1.5. Water samples were collected at equal time intervals up to 120 min of contact with AC6-600 within the pH range of 1–4, and the following graphs were obtained (Fig. 10b).

Surface oxygen functional groups on activated carbon are crucial for chromium sorption on activated carbon within the pH 2–4^77,71^. These functional groups possess pH-dependent charge, becoming more negative at higher pH values^66^. As a result, sorption of Cr(VI) ions should be minimal at low pH values, while generally decreases with increasing pH value from 5^78,64^, mainly via surface complexation and ion exchange between Cr(VI) ion and acidic functional groups^79,80^. Nevertheless, a general trend was observed for the removal efficiency of Cr(VI) ion, which decreased with increasing the pH of the media, recording its highest removal efficiency at pH 1, while the lowest one was at pH 11.3 (Fig. 10a). These results were consistent with those of other researchers^81,75,82^.

According to Attia et al.^83^, dichromate ions (Cr_2_O_7_^2–^) undergo reduction to Cr^3+^ in acidic conditions, while in basic conditions, it is oxidized, forming Cr(OH)_3_:

13 $${\rm{C}}{{\rm{r}}_{\rm{2}}}{\rm{O}}_7^{{\rm{2}} - } + {\rm{14}}{{\rm{H}}^ + } + {\rm{6e}} \to {\rm{2C}}{{\rm{r}}_{\rm{3}}}^{{\rm{2}} + } + {\rm{7}}{{\rm{H}}_{\rm{2}}}{\rm{O}}$$

14 $${\rm{C}}{{\rm{r}}_{\rm{2}}}{\rm{O}}_7^{2 - } + {\rm{4}}{{\rm{H}}_{\rm{2}}}{\rm{O}} + {\rm{3e}} \to {\rm{Cr}}{\left( {{\rm{OH}}} \right)_{\rm{3}}}~ + {\rm{5O}}{{\rm{H}}^ - }$$

Generally, Cr^3+^ exhibited a smaller trivalent cation than the uncharged Cr(OH)_3_; thus, Cr^3+^ is more readily adsorbed on AC600 than Cr(OH)_3_. Overall, rapid increases were observed in Cr ion removal efficiencies within the first 5 min of contact; thereafter, the changes became minimal, though still noticeable.

By combining chemical reduction and electrostatic attraction, nitrogen-doped activated carbon (AC600), which is made at 600 °C, efficiently eliminates Cr(VI) ions from aqueous solutions at acidic pH 1.5. At this pH, negatively charged Cr(VI) species such HCrO4⁻ and CrO4^2−^ are drawn to the positively charged surface of AC600 because of the protonation of nitrogen and oxygen functional groups. Then, with the help of surface functional groups and electron-donating nitrogen sites, Cr(VI) is reduced to less hazardous Cr(III). The amine (–NH_2_ to –NH_3_^+^) and carboxyl (–COOH) groups on AC600 are protonated by the high H^+^ concentration at pH 1.5, resulting in a positively charged surface. With removal efficiency frequently over 90%, this improves electrostatic interactions with anionic Cr(VI). When compared to undoped carbon, nitrogen doping amplifies protonation by increasing basic sites. By acting as electron donors, nitrogen atoms (pyridinic, pyrrolic, and graphitic) in AC600 reduce Cr(VI) to Cr(III) through the following reaction: HCrO_4_^–^ + 7 H^+^ + 3e^–^ → Cr^3+^ + 4H_2_O. Higher total Cr removal than Cr(VI) alone confirms that this uses H^+^ ions, increasing equilibrium pH. At pH 1.5, the procedure produces maximal capacity of about 245 mg/g. Because of electrostatic repulsion from the still-protonated surface, reduced Cr(III) either partially adsorbs onto residual oxygen/nitrogen sites or desorbs into solution. This dual fate explains why, at very low pH, Cr(VI) removal frequently outpaces total Cr removal. Overall efficiency is increased by nitrogen doping, which increases Cr(III) complexation.

### Regeneration of AC600

**Fig. 11:**
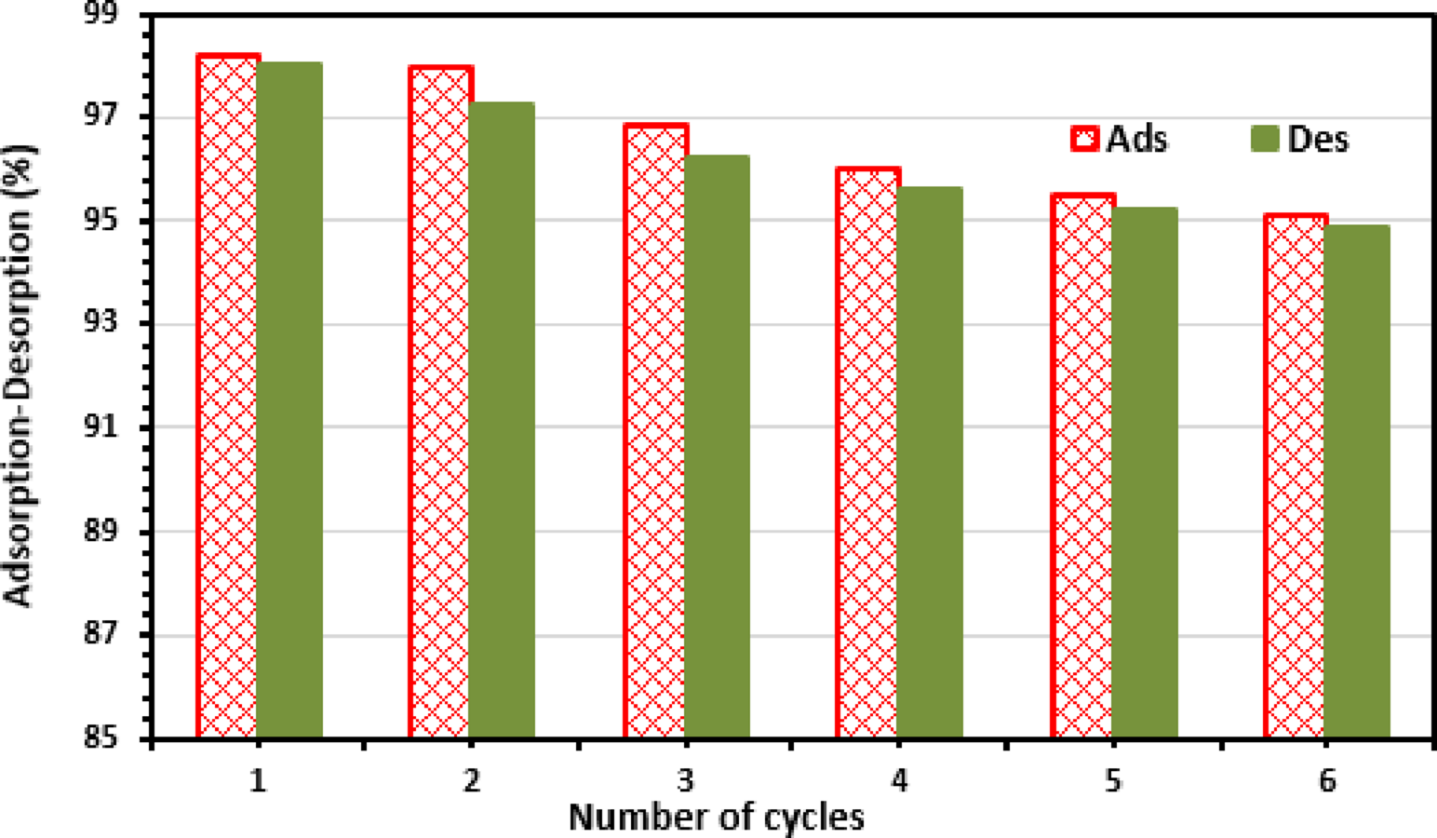
The effect of the regeneration rounds (cycles) on the removal percentage of Cr(VI) ion via desorption and adsorption % from the regenerated AC600 using 2.5 g L^–1^ of adsorbent dose and *C*_0_ of Cr(VI) ion of 100 mg L^–1^ at 25 ± 2 °C and pH 1.5 value.

Regeneration is a widely used method for removing wastewater from water that is solely dependent on reusing or recycling adsorbent components. The economic viability of this method is also heavily reliant on the reuse or recycling of these adsorbent components. Utilizing the chemical regeneration technique, the depleted active AC600 was restored. After being cleaned with 0.1 M NaOH solution and 0.1 M HCl solution, the activated AC600 was regenerated (recycled), washed with pure water, and dried. After six cycles of regeneration, the most significant amount of Cr(VI) ion that activated AC600 could remove was 95.12% (Fig. 11). This suggests that activated AC600 may be regenerated and utilized again without losing its adsorption ability^84^.

### RSM study

The chosen model was subjected to an ANOVA analysis of the Experimental design (Table 6) for Cr(VI) ion removal using AC600 adsorbent to determine its relevance and pinpoint the factors affecting the elimination percentage. Table 7 lists the ANOVA analysis results^28,29,30,31,32,33,36^. The F-values demonstrate the significance of the examined variables and how they interact with the selected response. The linked factor or interaction’s impact on the answer increases in significance as the F-value rises over 1. Based on the values shown in Table 7, it was found that the starting concentration of Cr(VI) ion had the greatest effect on the percentage of Cr(VI) ion eliminated. According to Table 7, the model’s F-value (29.50) indicates that it is crucial. Additionally, significant phrases are defined as those with *p*-values below 0.05. One may argue that the model’s relevance is shown by its *p*-value, which is less than 0.0001. The fact that the gap between the modified *R*^2^ (0.9975) and the expected *R*^2^ (0.9943) is less than 0.2 further demonstrates the validity of the model.

Based on the results obtained, the following Eqs. (15 and 16) for Cr(VI) ion removal % were obtained:

15 $$\begin{array}{ccccc}& {\rm{Removal }}\% {\rm{ for coded factors}} = {\rm{ 68}}.{\rm{686 }} + {\rm{ 8}}.{\rm{448A }} - {\rm{ 13}}.{\rm{165B }}\\& \quad + {\rm{ 3}}.{\rm{83}}0{\rm{C }} - {\rm{ }}0.{\rm{88}}0{\rm{A}} \times {\rm{B }} + {\rm{ }}0.{\rm{118A}} \times {\rm{C }} + {\rm{ 1}}.{\rm{1}}0{\rm{9B}} \times {\rm{C }} - {\rm{ }}0.{\rm{49}}0{{\rm{A}}^2}{\rm{ }}\\& \quad + {\rm{ 1}}.{\rm{967}}{{\rm{B}}^2}{\rm{ }} + {\rm{ 1}}.{\rm{741}}{{\rm{C}}^2}\end{array}$$

16 $$\begin{array}{ccccc}& {\rm{Cr}}\left( {{\rm{VI}}} \right){\rm{ionconc}}.){\rm{ }} + {\rm{ }}0.00{\rm{2 }}\left( {{\rm{dose X Time}}} \right){\rm{ }} + {\rm{ }}0.000({\rm{Cr}}\left( {{\rm{VI}}} \right){\rm{ionconc}}.{\rm{ X Time}}){\rm{ }}\\& \quad - {\rm{ }}0.{\rm{49}}0{\rm{ dos}}{{\rm{e}}^2}{\rm{ }} + {\rm{ }}0.000{\rm{Cr}}\left( {{\rm{VI}}} \right){\rm{ionconc}}{.^2}{\rm{ }} + {\rm{ }}0.00{\rm{1Tim}}{{\rm{e}}^2}\end{array}$$

**Table 6:** Experimental design for Cr(VI) ion removal using AC600 adsorbent.

Run	Factor 1 A: Dose (g L^–1^)	Factor 2 B: Cr(VI) Conc. (mg L^–1^)	Factor 3 C: Time (min)	Experimental removal %
1	0.5	250	60	58.36
2	0.5	400	120	56.23
3	1.5	400	90	59.25
4	1.5	200	15	71.84
5	1.5	100	60	83.43
6	1.5	100	60	83.43
7	0.5	100	15	73.21
8	2.5	100	120	98.05
9	2.0	250	60	72.73
10	0.5	100	120	78.39
11	2.5	400	15	61.33
12	0.5	100	120	78.39
13	2.5	200	15	79.47
14	2.5	400	90	67.16
15	0.5	400	60	49.75
16	2.5	100	120	96.25
17	0.5	400	120	55.63
18	1.5	250	120	74.66
19	0.5	100	15	73.21
20	1.0	400	15	50.08

**Table 7:** *F* and *p*-values determined by D-Optimal design for influential variables in Cr(VI) ion removal.

Source	Sum of squares	df	Mean square	F-value	*p*-value	
Model	3631.06	9	403.45	859.85	< 0.0001	Significant
A-Adsorbent dosage	753.82	1	753.82	1606.58	< 0.0001	
B-Cr(VI) ion Conc.	1909.41	1	1909.41	4069.41	< 0.0001	
C-Time	147.35	1	147.35	314.04	< 0.0001	
AB	6.76	1	6.76	14.40	0.0035	
AC	0.1077	1	0.1077	0.2296	0.6421	
BC	10.38	1	10.38	22.12	0.0008	
A^2^	0.7649	1	0.7649	1.63	0.2305	
B^2^	10.63	1	10.63	22.66	0.0008	
C^2^	9.89	1	9.89	21.07	0.0010	
Residual	4.69	10	0.4692			
Lack of fit	2.89	5	0.5784	1.61	0.3077	
Pure error	1.80	5	0.3600			
Cor total	3635.75	19				
Std. Dev.	0.6850	R^2^	0.9987			
Mean	71.04	Adjusted R^2^	0.9975			
C.V. %	0.9642	Predicted R^2^	0.9943			
Adeq precision	100.0477					

**Fig. 12:**
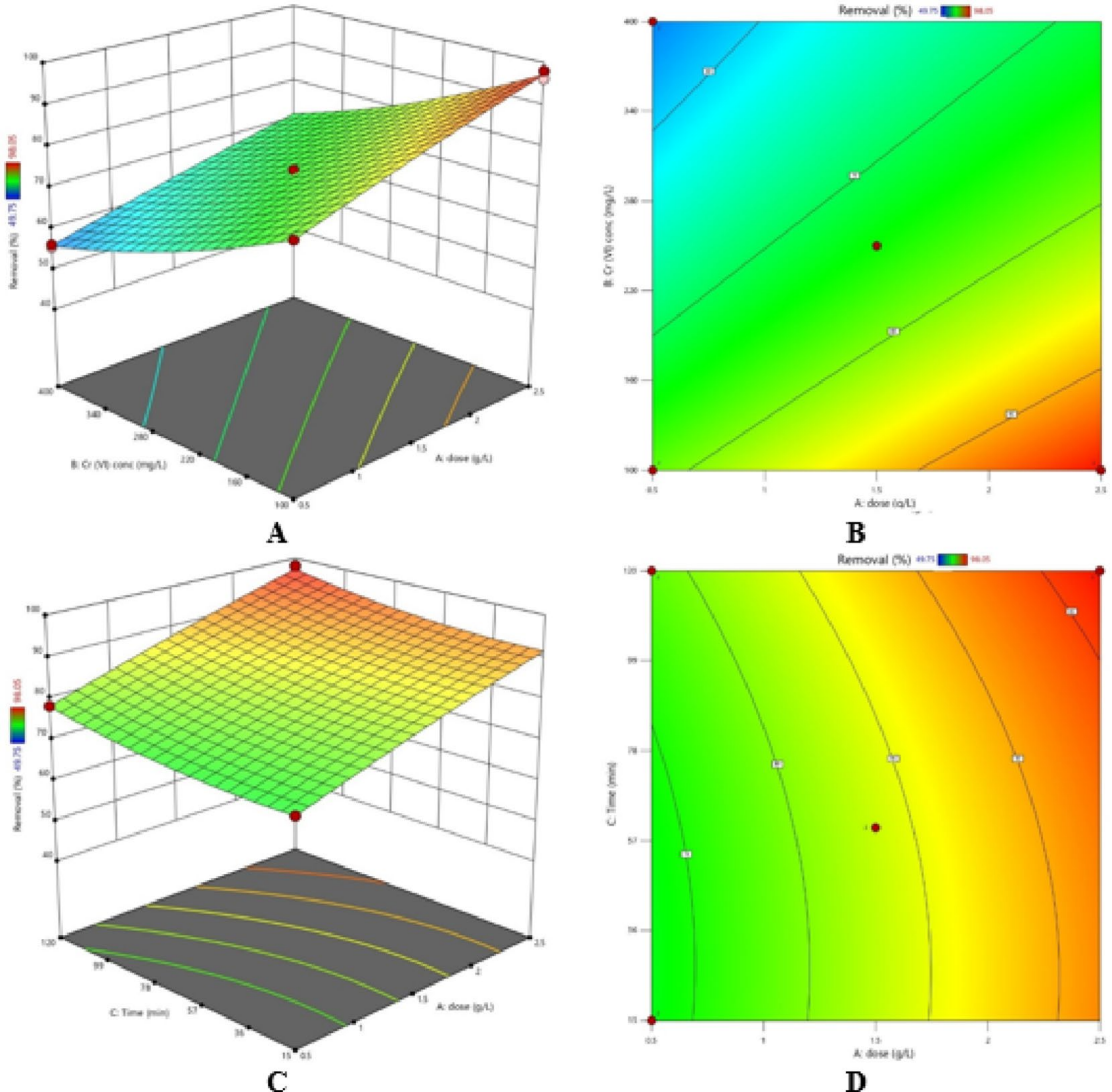

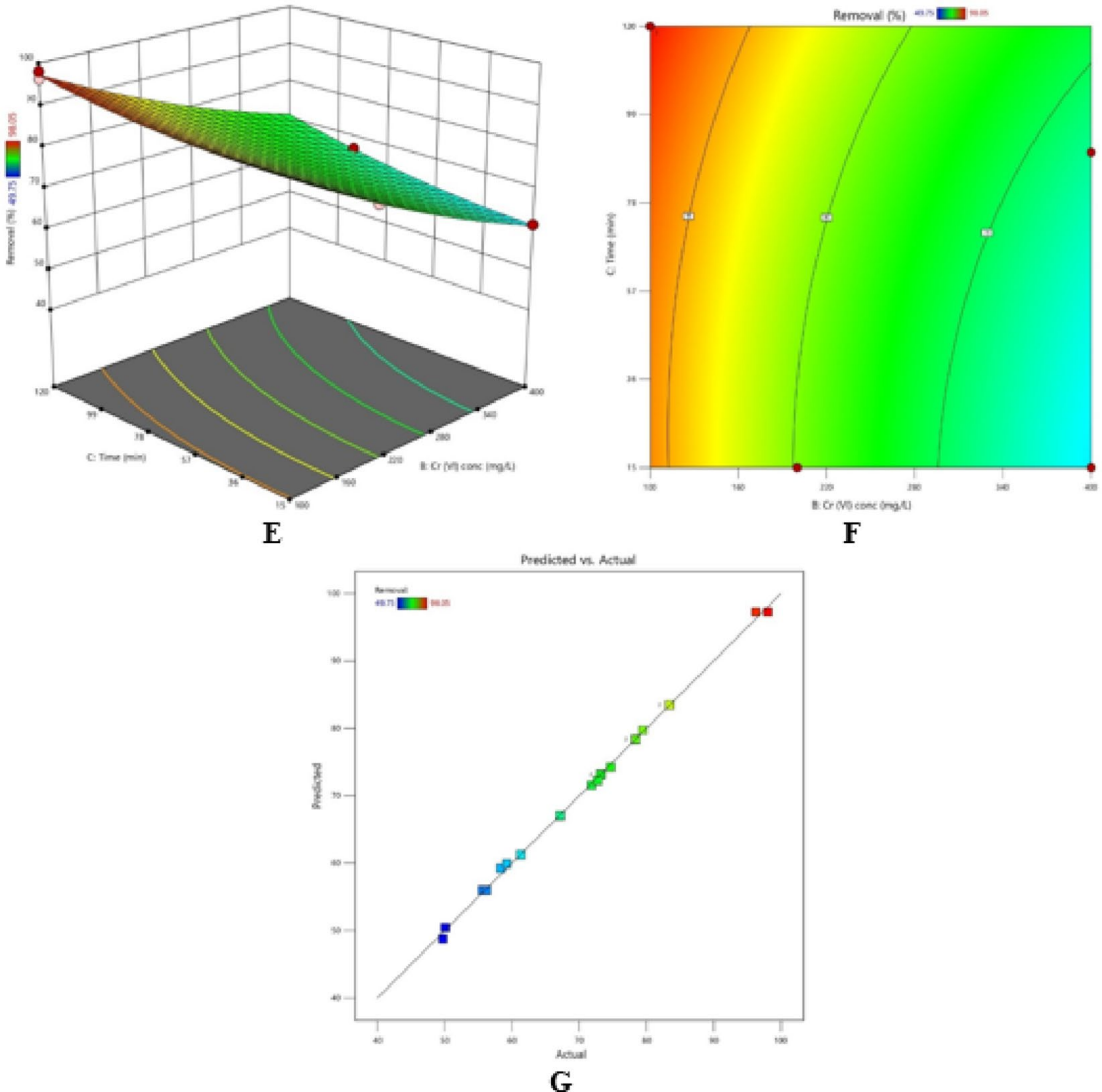
Combined effects of independent variables: (**A**,**B**) AC600 Adsorbent dose and Cr(VI) ion initial concentration, (**C**,**D**) AC600 Adsorbent dose and time, (**E**,**F**) Cr(VI) ion initial concentration and time and (**G**) predicted and actual values..

Figure 12 shows how the elimination % of Cr(VI) ions is affected by the combined impacts of reaction time, adsorbent dose, and starting Cr(VI) ion concentration. Additionally, Fig. 12 displays the actual and anticipated values. To get the highest removal %, low Cr(VI) ion concentrations, high AC600 adsorbent doses, and prolonged reaction times are optimal (Table 7)^34,35^.

**Fig. 13:**
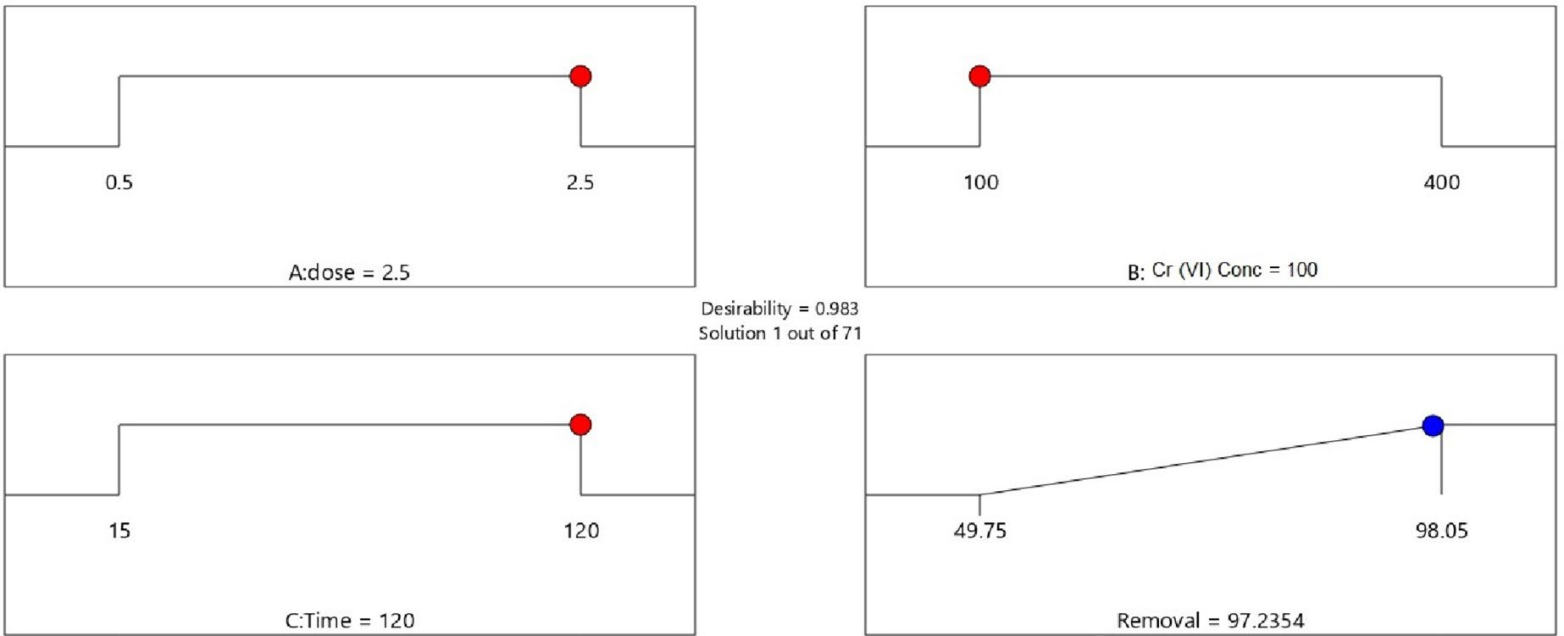
Optimization conditions through DOD settings.

The optimal operating settings were calculated numerically to provide the highest Cr(VI) ion removal %, as seen in Fig. 13.

### ANN modelling

**Fig. 14:**
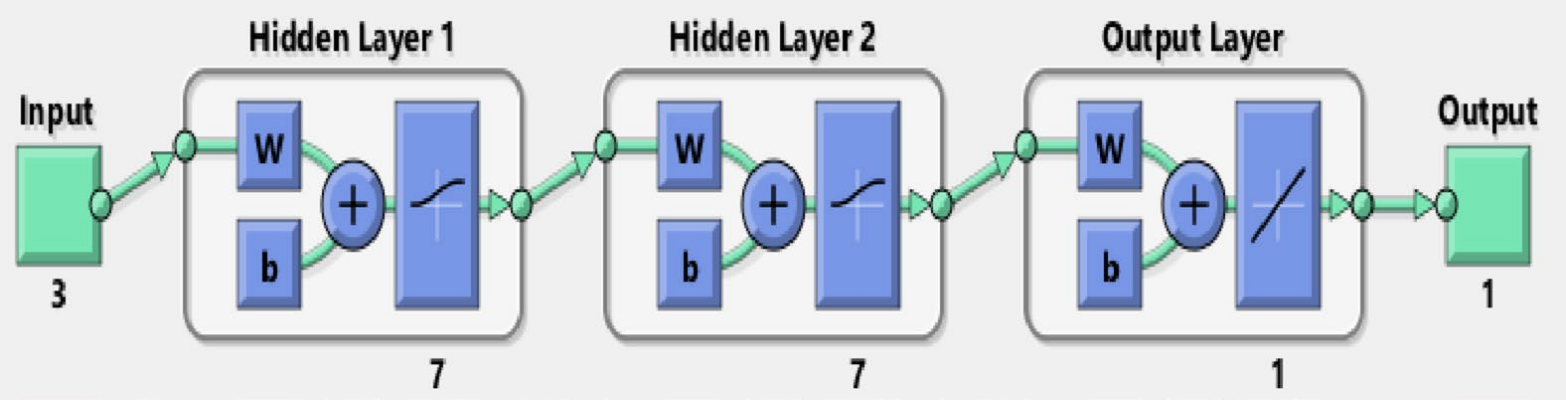
ANN architecture for the removal of Cr(VI) ions.

**Fig. 15:**
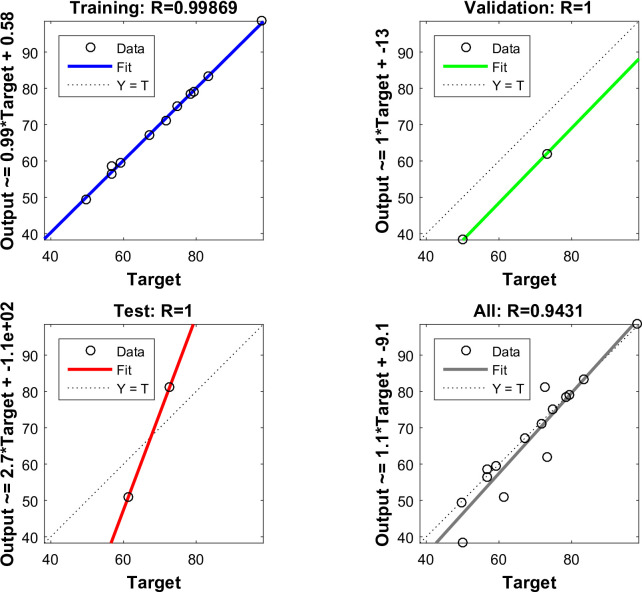
Datasets for the LM algorithm’s training, validation, testing, and general use.

**Fig. 16:**
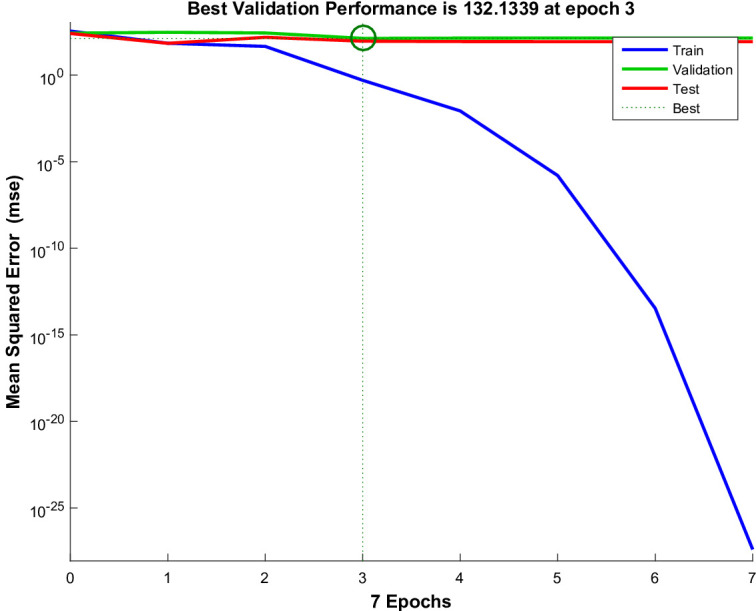
LM algorithm performance.

The removal of Cr(VI) by the AC600 ANN model was trained using the backpropagation algorithm with sample data divided into training (70%), testing (15%), and validation (15%). The optimal ANN model for removing Cr(VI) by the AC600 comprises 3 neurons in the input layer, 7 neurons in each of the two hidden layers, and 1 neuron in the output layer, as shown in Fig. 14. The regression plots in Fig. 15 showed that *R*^2^ training was 0.99869. *R*^2^ validation and testing were 1. *R*^2^ overall was 0.9431. The MSE value was 4.08e−28. The activation functions were log-sigmoid (log-sig) for each of the two hidden layers and purelin for the output layer. The optimal ANN input variables are the adsorbent dosage of AC600 (g/L), time (min), and initial concentrations of Cr(VI), while the output variable was the removal % of Cr(VI). Figure 16 displayed the MSE error vs. the epoch number for the optimized ANN model that stopped after 3 epochs^85,20,86,87^. The predicted results for RSM and ANN analyses are reported in Table 8.

**Table 8:** RSM and D-optimal ANN (removal of Cr(VI) ions using AC600).

Run	Factor 1	Factor 2	Factor 3	Response 1	Model prediction	
	A: dose	B: Cr ions conc	C: Time	Exp. Removal	RSM	ANN
	g L^–1^	mg L^–1^	min	%	%	%
1	0.5	250	60	56.66	59.25	56.66
2	0.5	400	120	56.63	55.99	57.07
3	1.5	400	90	59.25	59.92	59.25
4	1.5	200	15	71.84	71.57	71.84
5	1.5	100	60	83.43	83.47	84.52
6	1.5	100	60	83.43	83.47	84.52
7	0.5	100	15	73.21	73.14	73.42
8	2.5	100	120	98.05	97.24	98.05
9	2.0	250	60	72.73	72.27	73.41
10	0.5	100	120	78.39	78.34	78.39
11	2.5	400	15	61.33	61.25	61.33
12	0.5	100	120	78.39	78.34	78.39
13	2.5	200	15	79.47	79.71	79.47
14	2.5	400	90	67.16	67.05	67.16
15	0.5	400	60	49.75	48.78	49.75
16	2.5	100	120	98.05	97.24	98.05
17	0.5	400	120	56.63	55.99	57.10
18	1.5	250	120	74.66	74.26	74.66
19	0.5	100	15	73.21	73.14	73.42
20	1.0	400	15	50.08	50.44	50.08

### A comparative overview of Cr(VI) adsorption capacities

The *Q*_max_ of AC600 is compared to different adsorbent materials and reported in Table 9. Because of its increased microporosity and pyridine-like N-groups, which promote electrostatic attraction and redox reactions with Cr(VI) at pH 1.5, AC600 exhibits higher performance (245.44 mg/g). This confirms the effectiveness of our NH_3_-ZnCl_2_ dual pyrolysis approach by outperforming carbons from different sources.

**Table 9:** Comparison of Cr(VI) maximum adsorption capacities (*Q*_max_) for different adsorbent materials.

Material	Removal rate %	Q _m_ (mg/g)	Ref.
AC600	99	245.44	This work
*Chitosan* grafted crotonaldehyde	98.99	434.78	^88^
Red alga *Pterocladia capillacea*	58	12.85	^89^
Rubber	100	43.86	^90^
Microporous nano-activated carbon	99.12	13.33	^19^
Red algae (*Ceramium virgatum*)	90.0	26.5	^91^
Olive Leaves	~ 52.0	42.4	^92^
Activated magnetic biochar	99.7	9.97	^93^
AC from date palm seed	100	120.48	^94^
Wood bark-derived carbon	80.60	90	^95^
Biochar-SO	90.74	158.73	^13^
Watermelon peel biochar	69	72.46	^78^

## Conclusion

Nitrogen-doped activated carbon (AC600), synthesized via pyrolysis of organic residues with sawdust and ZnCl_2_ at 600 °C, exhibited high effectiveness for removal of Cr(VI) ion from wastewater. Its removal efficiency reached 95–99% when being used at 2.5 g L^–1^ especially at lower pH values. Its removal was characterized by a rapid rise within the first 20–40 min of contact, then these curves tend to equilibrate within 60–80 min. These curves sorption of Cr(VI) ion on AC600 which generally followed the Freundlich and Temkin isotherms, indicating multilayer adsorption on a heterogeneous surface. At low adsorbent doses, pseudo-second-order models were the best fitting kinetic model for Cr(VI) ion data, while power function and intraparticle diffusion models became more applicable at higher levels. Thereafter, AC600 was regenerated using sequential NaOH and HCl treatments to allow its repeated reuse. After 6 cycles, AC600 maintained high Cr(VI) removal efficiency of 95.12%, indicating that its adsorption capacity remained relatively stable. Ultimately, RSM optimization revealed that using 2.5 g of AC600 for decontamination of 100 mg L^–1^ Cr(VI) solution achieved the maximum removal efficiency of 97.24% within only 120 min^96,97,98,99,100,101,102,103,104,105,106,107^.

## Supplementary Information

Below is the link to the electronic supplementary material.

Supplementary Material 1

## Data Availability

Data sharing may be requested from the corresponding author, with an explanation of the reason.
