# Supplementary material for: Adsorptive removal of Cr(VI) ions using nitrogen-doping activated carbon: influence of pH, kinetics, isotherm models, optimization, and efficiency evaluation
Source: Sci Rep. 2026 Jun 11;16:18187. doi: 10.1038/s41598-026-53699-7 (PMC13260951; doi:10.1038/s41598-026-53699-7)
Supplement: Supplementary file 1 — Supplementary Material 1 [file 41598_2026_53699_MOESM1_ESM.docx]

**Supplementary material**

**Research Design**

**1 Research Approach**

This study used a laboratory-based experimental research approach to (i) synthesis and analyze nitrogen-doped activated carbon (AC600) from sawdust, and (ii) assess its efficacy in adsorbing and removing hexavalent chromium [Cr(VI)] from aqueous solutions.

The research integrates:

- Material synthesis and physicochemical characterization,
- Controlled batch adsorption experiments,
- Isotherm and kinetic modeling, and
- Process optimization and predictive modeling using Response Surface Methodology (RSM) and Artificial Neural Network (ANN).

The design is quantified, regulated, and reproduced to assure statistical dependability.

**2 Adsorbent Synthesis**

A preliminary experimental approach was employed to synthesis nitrogen-doped activated carbon (AC600) from sawdust through:

1. Pre-treatment
   - Drying at 105 °C to constant weight
   - Particle size standardization (1–2 mm)
2. Hydrothermal activation
   - Impregnation with ZnCl₂
   - Hydrothermal treatment at 180 °C for 5 h
3. Carbonization and Nitrogen Doping
   - Carbonization at 600 °C under NH₃ flow (100 mL/min) for 1 h
   - Tube furnace system with controlled gas flow
4. Post-treatment and purification
   - Acid washing (2N HCl)
   - Sonication
   - Ethanol washing and drying

The final product was labeled as AC600. This stage uses a controlled synthesis design in which temperature, activation duration, gas flow rate, and chemical impregnation ratio are fixed variables to produce a repeatable adsorbent.

**3 Characterization Design**

This characterization phase intended to correlate material attributes with adsorption performance. A descriptive analytical design was employed to determine the physicochemical properties of AC600 using:

- Surface morphology & elemental composition: SEM–EDX
- Surface functional groups: FTIR
- Surface area & pore structure: BET and BJH (N_2_ adsorption at 77 K)
- Crystallinity: XRD
- Thermal stability: TGA (25–1000 °C, N_2_ atmosphere)
- Surface chemical states: XPS

**4 Batch Adsorption Design**

A controlled batch adsorption experimental approach was employed to investigate Cr(VI) removal performance.

Independent Variables:

- Adsorbent dose (0.5–2.5 g/L)
- Initial Cr(VI) concentration (100–400 mg/L)
- Contact time (10–120 min)
- Solution pH (1.5–11.3)

Dependent Variables:

- Removal efficiency (RE%)
- Adsorption capacity (qₑ, mg/g)

Controlled Variables:

- Temperature (25 ± 2 °C)
- Shaking speed (200 rpm)
- Solution volume (100 mL)
- Synthetic Cr(VI) solution in deionized water

Experiments were conducted in triplicate to ensure reproducibility (standard deviation ≤ 2.2). Cr(VI) concentrations were measured spectrophotometrically at λ_545_ nm using 1,5-diphenylcarbazide. A control sample (without adsorbent) was used to confirm Cr(VI) stability and eliminate experimental bias.

**5 Adsorption Modeling Design**

A comparative mathematical modeling design was employed to interpret adsorption mechanisms.

Isotherm Models Applied: These theories explain adsorption equilibrium and surface heterogeneity.

- Langmuir
- Freundlich
- Temkin
- Halsey

Kinetic Models Applied: Model fitting was tested using statistical parameters and the standard error of estimations.

- Pseudo-first-order
- Pseudo-second-order
- Elovich
- Power function
- Intraparticle diffusion

**6 Response Surface Methodology (RSM)**

A multivariate optimization design utilizing Response Surface Methodology (RSM) was implemented.

- Software: Design-Expert v13
- Number of experiments: 20
- Design type: D-Optimal Design (DOD)

Factors:

- A: Adsorbent dose (0.5–2.5 g/L)
- B: Contact time (15–120 min)
- C: Initial Cr(VI) ions concentration (100–400 mg/L)

Response:

- Cr(VI) ions removal efficiency (%)

RSM was used to:

- Determine interaction effects
- Develop a predictive polynomial model
- Identify optimal operating conditions

**7. Artificial Neural Network (ANN)**

A computational predictive modeling architecture was utilized to simulate and predict Cr(VI) ions removal performance.

- Software: MATLAB R2015b
- Algorithm: Levenberg–Marquardt (LM)
- Data division:
  - Training: 70%
  - Validation: 15%
  - Testing: 15%

**ANN Structure:**

- Input layer:
  - Adsorbent dose
  - Contact time
  - Initial Cr(VI) concentration
- Two hidden layers (7 neurons each)
- Output layer: Cr(VI) removal efficiency (%)

ANN modeling was used to:

- Capture nonlinear relationships
- Improve prediction accuracy
- Compare performance with RSM

The combination of experimental inquiry with statistical and machine learning methodologies enables both mechanistic understanding and predictive capability for Cr(VI) removal utilizing nitrogen-doped activated carbon (AC600).
